# Surveillance-Driven Machine Learning for Prediction of Antimicrobial Susceptibility: An Explainable Modeling Framework using the Pfizer ATLAS Dataset (2004 - 2023)

**DOI:** 10.12688/wellcomeopenres.26477.1

**Published:** 2026-06-27

**Authors:** Raphael Mutua, David Gichohi, Samuel K. Ndegwa, Rahma O. Golicha, Frida Njeru, Benson Kituku

**Affiliations:** 1 Center for Microbiology Research, Kenya Medical Research Institute, Nairobi, Nairobi County, 54840-00200, Kenya; 2 Department of Computer Science, Dedan Kimathi University of Technology, Nyeri, Nyeri County, 657-10100, Kenya

**Keywords:** Antimicrobial Resistance, Machine Learning, AMR Surveillance, Predictive Modelling, Antimicrobial Susceptibility

## Abstract

**Background:**

Antimicrobial resistance (AMR) is currently a major global health problem, with Sub-Saharan Africa bearing the brunt of the burden. This heavy burden of AMR is mainly due to poor diagnostic infrastructure and limited Antimicrobial Stewardship (AMS) programs. Timely pathogen detection and Antimicrobial Susceptibility Testing (AST) are crucial for improving patient health outcomes. Conventional culture-based AST methods take 48–72 hours, raising the need for immediate empirical antibiotic therapy. This calls for the development of rapid and easy-to-use techniques for AMR prediction and monitoring for routine and epidemiological surveillance.

**Methods:**

We developed surveillance-driven Machine Learning (ML) models using the Pfizer Antimicrobial Testing Leadership and Surveillance (ATLAS) dataset (2004–2023). The study integrated a rule-based Minimum Inhibitory Concentration (MIC) interpretive tool with antibiotic-specific predictive models. It also incorporated an interactive dashboard for data visualization and decision support. Model performance was evaluated using training and testing accuracy, alongside robustness assessments.

**Results:**

The predictive models demonstrated testing accuracies ranging between 57% and 88% across antibiotics. Bacterial families and species were the best predictors of antibiotic susceptibility, with geographic location, specimen source, and clinical specialty also contributing significantly. In contrast, demographic variables showed comparatively lower predictive value.

**Conclusion:**

Routinely collected AMR surveillance data can be leveraged to develop interpretive and scalable predictive models. Such models are more compatible with existing surveillance infrastructure and may be integrated into clinical workflows with relatively minimal additional resource requirements.

## Plain Language summary

Antimicrobial resistance (AMR) occurs when bacteria no longer respond to medicines used to treat infections, making illnesses harder to treat and increasing the risk of severe outcomes. This is a growing global health problem, particularly in Low- and Middle-Income Countries (LMICs) where access to rapid diagnostic testing and Antimicrobial Stewardship (AMS) programs may be limited.

In many healthcare settings, laboratory testing to determine which antibiotics are effective can take 48–72 hours. During this time, patients are often treated with broad-spectrum antibiotics without knowing whether they will work. This can lead to unnecessary antibiotic use, higher treatment costs, and the development of further resistance.

This study explored whether routinely collected AMR surveillance data can be used to predict antibiotic effectiveness. Using data from the Pfizer ATLAS surveillance program, we developed computer-based models that estimate the likelihood that a specific antibiotic will be effective against a bacterial infection. These predictions are based on factors such as the type of bacteria, geographic location, and sample source.

We also developed a rule-based tool that interprets laboratory measurements into standard susceptibility categories and an interactive dashboard that allows users to explore resistance trends and model predictions in a user-friendly way.

The results show that surveillance data can be used to generate meaningful predictions about antibiotic effectiveness, even without advanced laboratory or genomic data. These tools could support earlier and more informed treatment decisions, improve antibiotic use, and strengthen monitoring of resistance patterns.

However, the accuracy of these predictions depends on the quality and completeness of available data, and further validation is needed before routine clinical use.

Overall, this study demonstrates how existing surveillance data can be transformed into practical tools to support better use of antibiotics and help slow the spread of antimicrobial resistance, particularly in resource-limited settings.

## Introduction

Antimicrobial Resistance (AMR) has emerged as one of the defining public health challenges of the twenty-first century ( Baekkeskov & Pierre, 2024). According to the Global Burden of Disease, bacterial AMR was associated with 4.95 million global deaths in 2019, with 1.27 million deaths being directly attributable to resistant infections ( Murray et al., 2022). This presents a significant global health challenge, with Sub-Saharan Africa bearing a disproportionate burden ( Kariuki et al., 2022). These findings highlight the urgent need for improved surveillance, diagnostics, and stewardship strategies capable of mitigating the spread of resistant pathogens ( Ahmed, 2025).

Timely pathogen detection and Antimicrobial Susceptibility Testing (AST) are central to effective therapy. Conventional culture-based AST methods typically require 48–72 hours, during which empirical antibiotic therapy is initiated ( Syal et al., 2017). During this interval, clinicians must initiate empirical antibiotic therapy, often relying on broad-spectrum agents to ensure adequate coverage. In many Low- and Middle-Income Countries (LMICs), where diagnostic infrastructure and Antimicrobial Stewardship (AMS) programs may be limited, empirical prescribing often involves broad-spectrum agents, contributing to resistance selection ( Reali et al., 2025).

Beyond its clinical consequences, delayed susceptibility results also have economic implications. Empirical broad-spectrum therapy is associated with increased drug expenditure, prolonged hospital stays, and a higher risk of treatment failure or complications requiring additional interventions ( Lucidi et al., 2017). These downstream effects collectively impose substantial costs on already constrained healthcare systems, particularly in LMICs ( Sulis et al., 2022).

Global surveillance platforms have been established to monitor emerging resistance patterns and guide public health ( Jia et al., 2023). Platforms such as the World Health Organization Global Antimicrobial Resistance Surveillance System (GLASS) compile susceptibility data from participating laboratories across multiple regions ( WHO, 2023). These datasets provide valuable epidemiological insights into the geographic distribution of resistance across pathogens and antimicrobial agents. However, surveillance datasets are often analyzed descriptively rather than being used as predictive tools capable of informing clinical decision-making.

Recent advances in Machine Learning (ML) have demonstrated substantial potential for predicting antimicrobial resistance phenotypes ( Kim et al., 2022). Many ML approaches rely on genomic sequencing data, which can capture genetic determinants of resistance with a high predictive accuracy ( Kim et al., 2022; Ren et al., 2022). However, genomic prediction frameworks require specialized sequencing infrastructure, bioinformatics expertise, and significant computational resources. These requirements limit their scalability in many LMIC contexts where genomic sequencing capacity remains unevenly distributed ( Nkhono et al., 2026; Olorunkosebi et al., 2025).

Consequently, there is growing interest in alternative modeling approaches that leverage routinely collected surveillance metadata rather than genomic features ( Chindelevitch et al., 2022). Epidemiological variables such as pathogen species, geographic locations, specimen source, and temporal trends may capture underlying resistance ecology and therefore provide useful predictive signals. Models based on these variables may be more readily deployable in routine clinical settings, particularly in resource-constrained environments where microbiological and epidemiological data are available but genomic sequencing is not.

The Pfizer Antimicrobial Testing Leadership and Surveillance (ATLAS) program provides one of the most comprehensive global datasets for AMR surveillance. The ATLAS database compiles standardized susceptibility testing data from participating laboratories worldwide and spans more than two decades of resistance monitoring ( Leclerc et al., 2020). The dataset includes Minimum Inhibitory Concentration (MIC) measurements, susceptibility interpretations, pathogen identification, specimen type, and country, making it very suitable for data-driven modeling approaches.

In addition to its breadth of coverage, the longitudinal nature of the ATLAS dataset offers an opportunity to explore temporal dynamics of resistance and to develop predictive frameworks that account for evolving epidemiological patterns. Such analyses can support both clinical decision-making and broader public health planning by identifying emerging resistance trends and informing stewardship interventions.

Despite the availability of extensive global surveillance datasets, relatively few studies have explored their potential for predictive modeling of antimicrobial susceptibility. This represents a critical opportunity to enhance the practical utility of surveillance systems.

To address these challenges, this study integrates interpretive breakpoint translation with antibiotic-specific ML prediction using ATLAS data within a One Health-informed stewardship framework.

This study specifically aimed to;

1. Develop a rule-based MIC interpretive engine capable of translating raw MIC measurements into standardized susceptibility categories based on established clinical breakpoints.
2. Train antibiotic-specific ML models to predict susceptibility using structured surveillance metadata.
3. Evaluate model performance using robust statistical metrics, including Area Under the Receiver Operating Characteristic Curve (AUROC), accuracy, precision, and recall.
4. Apply SHapley Additive exPlanations (SHAP) methods to assess model interpretability and identify key predictors influencing resistance predictions.
5. Provide open-source, reproducible tools that can support AMS and surveillance initiatives.

By combining interpretive algorithms with explainable machine learning tools, this work aims to demonstrate how routinely collected surveillance data can be transformed into decision-support tools capable of informing empirical antibiotic prescribing, improving surveillance analytics, and strengthening AMS strategies in resource-limited settings.

## Methods

### Study design and data source

This study was designed as a retrospective secondary analysis of global antimicrobial resistance surveillance data derived from the Pfizer ATLAS program. The ATLAS database compiles standardized antimicrobial susceptibility testing results from participating laboratories across multiple continents, spanning the period 2004–2023.

The dataset includes organism-level MIC measurements, interpreted susceptibility classifications, patient demographic characteristics, clinical source information, and geographic identifiers. This data is integrated using standardized laboratory methodologies and centralized quality control procedures, aligned with Clinical and Laboratory Standards Institute (CLSI) or European Committee on Antimicrobial Susceptibility Testing (EUCAST) guidelines, enabling cross-site comparability of susceptibility results.

For the predictive modeling component, we intentionally restricted analyses to African isolates collected between 2019 and 2023. Resistance patterns exhibit substantial geographic heterogeneity and may evolve rapidly over time. Limiting the dataset to recent isolates from a specific region improves the contextual relevance and reduces the influence of historical resistance dynamics that may no longer reflect current epidemiological conditions.

Focusing on recent African data also aligns with the broader objective of developing predictive tools that are applicable within LMIC contexts. The epidemiology of AMR differs significantly between regions due to variations in antibiotic use patterns, healthcare infrastructure, infection prevention practices, and regulatory environments. Models trained on globally aggregated datasets may therefore perform poorly when applied to specific regional contexts. By restricting training data to African isolates, this study aimed to enhance the external validity of predictions for stewardship applications in Sub-Saharan Africa (SSA).

All analyses were conducted using Python (v3.10) within a reproducible computational environment. The modeling workflow incorporated standard data preprocessing steps, including missing-value handling, categorical feature encoding, and normalization where appropriate. Feature engineering was performed to transform raw surveillance variables into structured predictors suitable for machine learning algorithms. Model development and evaluation were conducted using the Extreme Gradient Boosting (XGBoost).

To ensure reproducibility, the computational environment was containerized, and all analyses were version-controlled using Git. This was done to facilitate independent verification of the modeling workflow and support transparent reporting of the procedures.

### Data preprocessing and inclusion criteria

The Pfizer Atlas antibiotics dataset had data collected between 2004 and 2023 across 83 countries in the world. It comprised over 960,000 bacterial isolates.

To develop a predictive model, we used a subset of this dataset. The subset was extracted based on temporal and geographic relevance to ensure contextual applicability. We focused on isolates between 2019 and 2023 from African countries.

In data processing, we first excluded records with missing and inconsistent entries. To ensure compatibility with machine learning algorithms, we standardized and encoded categorical variables. We retained MIC values for the interpretive model for susceptibility categories: Susceptible, Intermediate, and Resistant.

The inclusion criteria for the antibiotics in the model were based on sufficient susceptible instances in the dataset. This was to ensure our model remains stable. This resulted in the inclusion of commonly used and clinically relevant antibiotics such as Gentamicin, Amikacin, Imipenem, and Ceftazidime, among others.

### Development of an interactive dashboard

We developed an integrated, web-based interactive dashboard for the analysis, model output, and visualization of AMR resistance trends ( Figure 1). The dashboard enabled users to:

1. Explore AMR trends across countries, organisms and antibiotics
2. Interact with an interpretive model that translates MIC values into clinical susceptibility categories based on organism, specimen source, and antibiotic class ( Figure 2).
3. Engage with a predictive model to identify the most effective antibiotic based on patient and isolate’s characteristics, including species, family, country, gender, age group, clinical specialty, and specimen source ( Figure 3).

**Figure 1.**
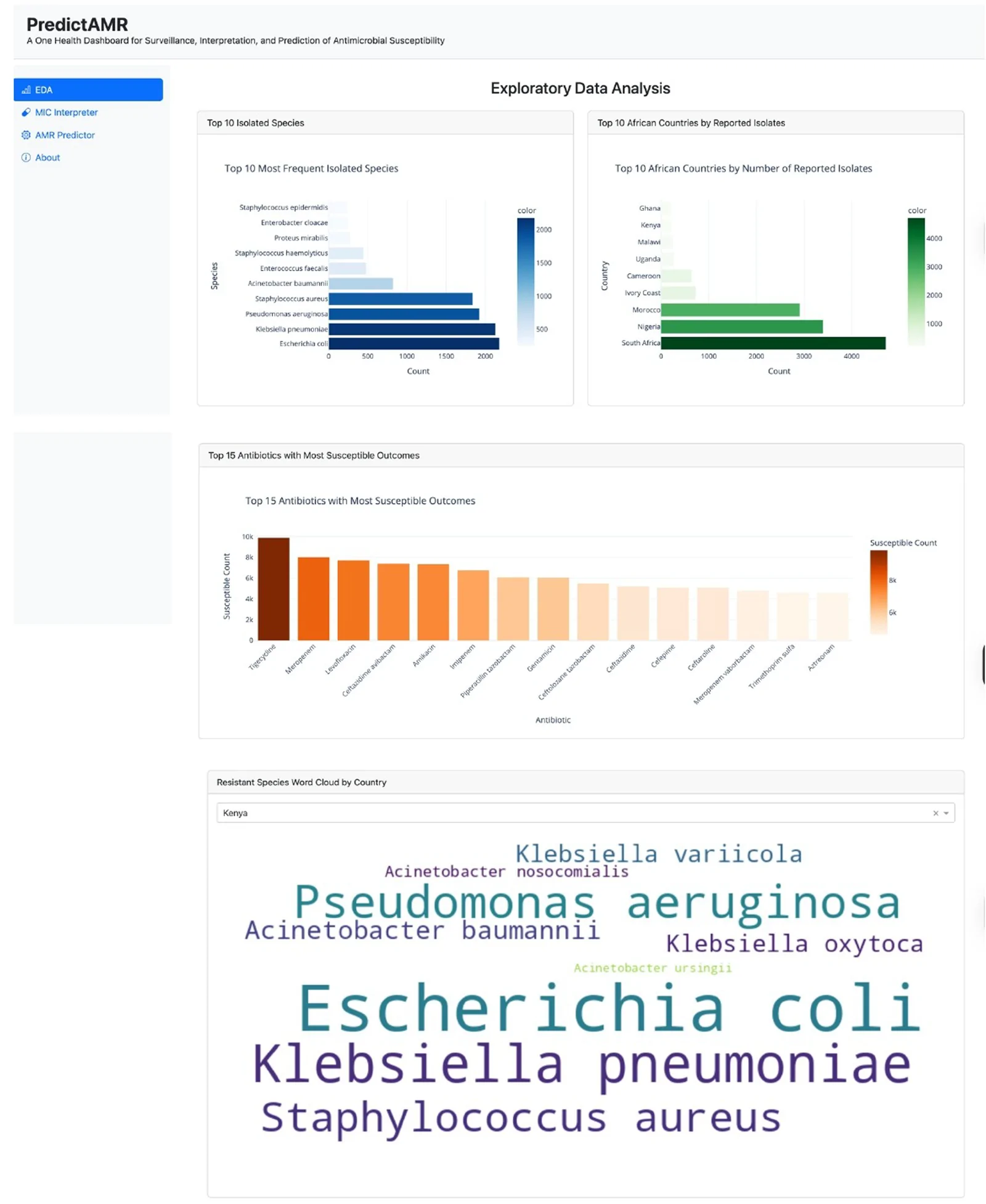
Interactive dashboard for AMR surveillance and prediction. The interface allows users to explore AMR trends across countries, organisms, and antibiotics through dynamic filtering and visualization tools.

**Figure 2.**
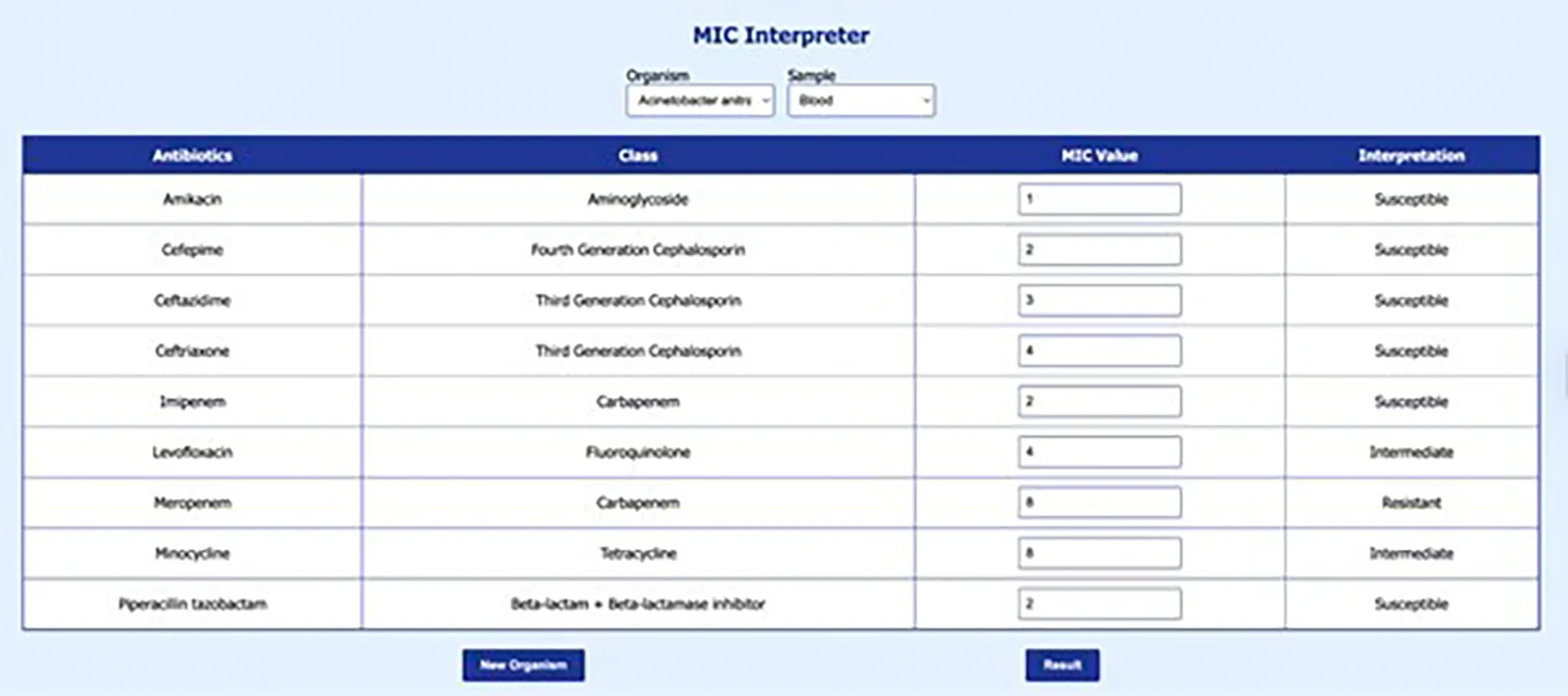
Rule-based MIC interpretive tool for susceptibility classification. The tool converts MIC values into standardized susceptibility categories based on organism, specimen source, and antibiotic class.

**Figure 3.**
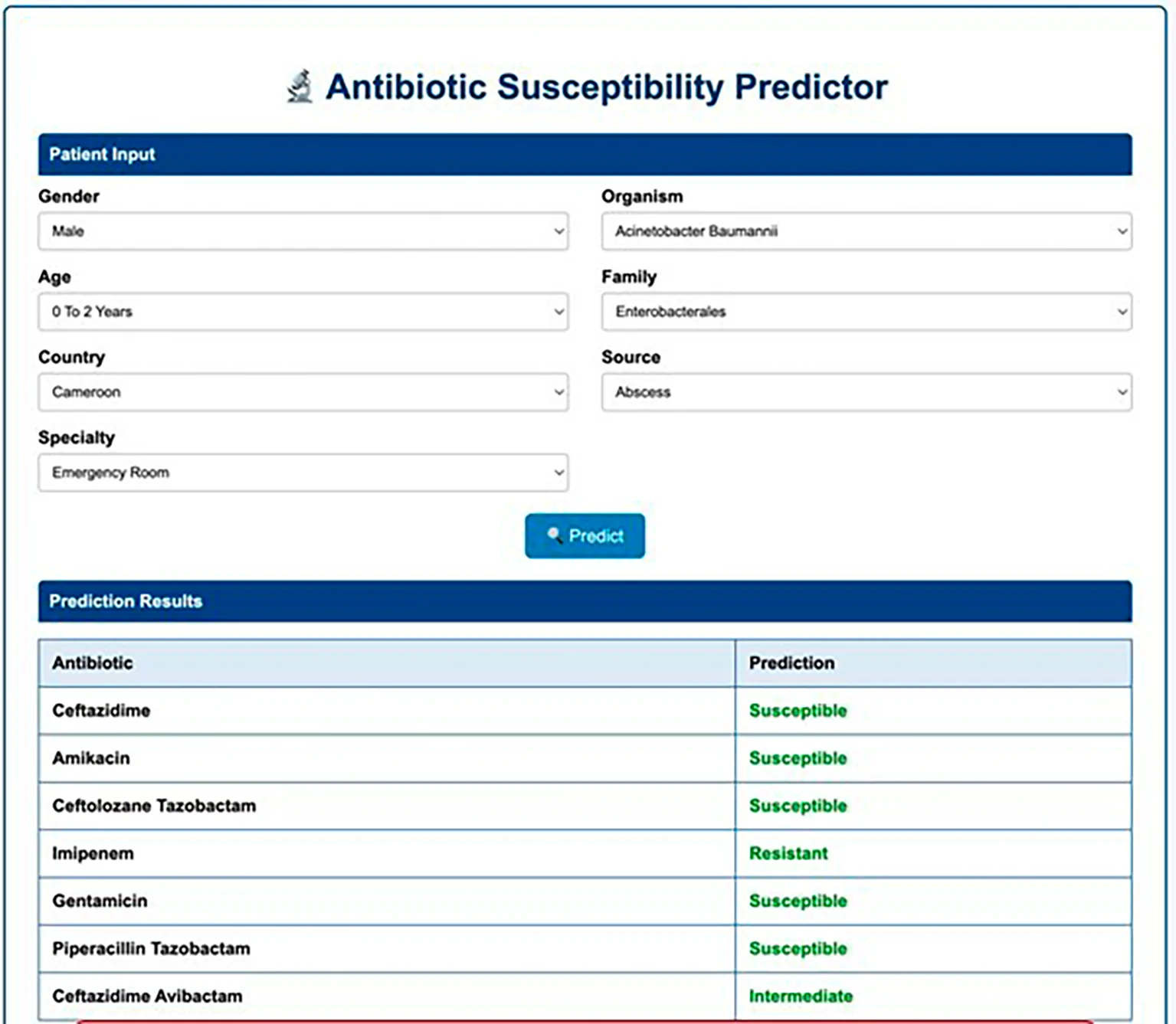
Machine learning model for antimicrobial susceptibility prediction. The model generates probabilistic predictions of antibiotic effectiveness based on epidemiological and clinical input variables.

The dashboard served as a central interface for surveillance-driven decision-making, enabling clinicians, researchers, and policymakers to derive actionable insights from complex AMR datasets.

### Development of the interpretive tool

The interpretive tool ( Figure 2) was developed as a rule-based system to translate MMIC values into clinical susceptibility categories (Susceptible (S), Intermediate (I) and Resistant (R)) based on the organism, sample source, and antibiotic class.

The interpretive rules were derived from the dataset by mapping MIC thresholds to susceptibility outcomes based on organism type, antibiotic class, and specimen source. These rules were encoded into structured JavaScript Object Notation (JSON) format and implemented using Python.

Integrated within the dashboard, the tool guided the user through a structured interpretation workflow. Upon entry of the organism name, the system dynamically displayed a list of antibiotics recommended for that organism in accordance with Clinical and Laboratory Standards Institute (CLSI) guidelines. Users then input the corresponding MIC values, after which the system automatically generated susceptibility interpretations for the selected antibiotics. This streamlined process supported standardized, accurate, and rapid clinical decision-making.

### Machine learning model development

We developed and trained seven antibiotic-specific models ( Figure 3) using the XGBoost ensemble algorithm, focusing on antibiotics with the highest proportion of susceptible cases: Gentamicin, Ceftazidime-avibactam, Amikacin, Imipenem, Piperacillin-tazobactam, Ceftolozane-tazobactam, and Ceftazidime. The input features included bacterial species, family, country, gender, age group, and specimen source, among others.

Pre-modeling exploratory analyses identified key resistant species and country-level variability in resistance patterns. Hyperparameter tuning was performed to optimize the model performance. Model evaluation metrics considered both training and testing accuracy; however, class imbalance (S/I/R) affected generalization performance, especially for underrepresented classes.

### Model evaluation and statistical analysis

The primary evaluation metrics were model accuracy, training accuracy, and test accuracy. Accuracy was defined as the proportion of correctly classified instances across susceptibility categories.

In addition, model performance was assessed using precision, recall, and AUROC to provide a more comprehensive evaluation across classification thresholds.

Model robustness was assessed using comparative performance across antibiotics, sensitivity to class distribution, and consistency between training and testing results.

Class imbalance across susceptibility categories was explicitly considered when interpreting performance metrics, particularly for underrepresented classes.

## Results

### Dataset characteristics

The dataset is one of the most comprehensive global AMR repositories, with isolates spanning multiple continents and years. It included diverse bacterial species and antibiotic susceptibility profiles, with variability across countries and specimen sources.

It was highly imbalanced, where susceptible isolates had higher proportions than intermediate and resistant categories.

### Model performance

The predictive models demonstrated variable performance across antibiotics, reflecting differences in data distribution and complexity of resistance patterns. Testing accuracies ranged from 57% to 88%. The training and testing accuracies of the models are shown in Table 1.

**Table 1.** Performance metrics of antibiotic-specific machine learning models.

Model	Susceptible instances	Training accuracy	Testing accuracy
Tigecycline_XGB	9890	95%	88%
Meropenem_I	8036	83%	71%
Levofloxacin_I	7720	70%	58%
Ceftazidime avibactam_I	7394	86%	76%
Amikacin_I	7339	76%	57%
Imipenem_I	6787	78%	64%
Piperacillin tazobactam_I	6101	73%	62%
Gentamicin_I	6080	79%	65%
Ceftolozane tazobactam_I	5522	83%	62%
Ceftazidime_I	5211	74%	62%

### Model interpretability using SHapley Additive exPlanations (SHAP)

To enhance the transparency and interpretability of the machine learning models, SHAP was employed to quantify feature contributions to model predictions.

SHAP analysis revealed that:

1. Bacterial species and family were the most influential predictors of antibiotic susceptibility.
2. Geographic location (country) significantly influenced resistance patterns, reflecting regional variability.
3. Specimen source and clinical specialty contributed to prediction outcomes, indicating context-specific resistance trends.
4. Demographic factors such as age group and gender had a relatively lower but still notable influence.

This interpretability framework strengthens the applicability of the model in real-world clinical and surveillance settings.

### Conceptual framework for surveillance-driven prediction

The study proposes a surveillance-driven prediction framework that integrates retrospective AMR data, machine learning models, and interactive visualization tools into a unified decision-support system.

The framework is structured into four layers: data, processing, modeling, and application.

1. Data Layer—Large-scale AMR datasets are collected from multiple sources, including clinical laboratories, surveillance networks, and public repositories.
2. Processing Layer—The collected data undergoes cleaning, filtering, and feature engineering to ensure quality and suitability for modeling.
3. Modeling Layer—Both interpretive and predictive models are developed.
4. Application Layer—The processed data and model outputs are delivered through an interactive dashboard, enabling clinicians, researchers, and policymakers to access actionable insights in real-time.

This layered framework facilitates evidence-based decision-making across clinical and public health settings.

## Discussion

### Principal findings

This study demonstrates that routinely collected AMR surveillance data can be leveraged to build both interpretive and predictive tools that are potentially deployable in resource-limited settings. Unlike genomic machine-learning models that require sequencing infrastructure, the approach presented here operates on epidemiological metadata derived from routine surveillance systems, enhancing scalability and practical applicability.

The dual-model approach developed in this study addresses two complementary challenges in AMS.

First, the MIC interpretation engine provides a systematic method for translating raw laboratory measurements into standardized susceptibility categories. Such tools, based on current guidelines, can help harmonize susceptibility interpretation across datasets and support automated data processing in surveillance pipelines.

Second, the machine learning prediction models generate probabilistic estimates of antimicrobial susceptibility before laboratory confirmation. These predictions could potentially inform empirical prescribing decisions during the period between specimen collection and the availability of AST results.

Importantly, the integration of explainable machine learning methods provides transparency regarding the variables driving model predictions. This feature is particularly important in a clinical decision-support context, where interpretability is necessary to foster trust among clinicians and policymakers.

### Comparison with existing literature

Previous studies have demonstrated the potential of machine learning approaches for predicting AMR using genomic features such as k-mer frequencies and resistance gene profiles ( Kim et al., 2022; Ren et al., 2022). These models often achieve high predictive performance because they directly capture genetic determinants of resistance mechanisms.

However, genomic approaches also present significant implementation challenges. Whole-genome sequencing requires specialized laboratory equipment, bioinformatics expertise, and computational infrastructure that may not be readily available in many healthcare settings, particularly in LMICs ( Olorunkosebi et al., 2025).

In contrast, models based on surveillance metadata can be developed using routinely collected clinical and epidemiological information. Such models are therefore more compatible with existing surveillance infrastructure and may be integrated into clinical workflows with relatively minimal additional resource requirements.

Recent work has begun exploring this paradigm, demonstrating that epidemiological predictors such as geographic location, pathogen species, and specimen source can provide meaningful predictive signals for resistance phenotypes ( Chindelevitch et al., 2022). This study contributes to this emerging literature by applying these principles to a large global dataset and focusing specifically on the African context.

### Biological and epidemiological implications

The prominence of geographic location and pathogen species as important predictors in SHAP analyses highlights the spatial and ecological heterogeneity of AMR.

Resistance patterns are shaped by multiple interacting factors, including local antibiotic consumption practices, infection prevention policies, environmental reservoirs of resistance genes, and patterns of healthcare utilization ( Hossain & Chowdhury, 2024; Ye et al., 2025). As a result, the prevalence of resistance may vary substantially even between neighboring countries or healthcare systems ( Coque et al., 2023).

The strong influence of geographic variables observed in this study underscores the importance of context-specific surveillance and modeling. Predictive tools that fail to account for regional variation may produce misleading estimates of susceptibility and could inadvertently contribute to inappropriate prescribing decisions.

The comparatively lower predictive performance observed for fluoroquinolone resistance may reflect the complex genetic mechanisms underlying resistance to this class of antibiotics. Fluoroquinolone resistance can arise through multiple pathways, including chromosomal mutations in target genes and plasmid-mediated resistance determinants. These mechanisms may spread rapidly across bacterial populations, leading to dynamic resistance patterns that are difficult to capture using static surveillance features.

Future modeling approaches may therefore benefit from incorporating temporal variables or time-series frameworks capable of capturing evolving resistance trends.

### Public health and policy implications

Predictive AMR models derived from surveillance data have several potential applications in public health and AMS.

First, they may support empirical antibiotic selection in clinical settings where laboratory results are not immediately available. Even modest improvements in the accuracy of empirical prescribing could reduce inappropriate use of broad-spectrum antibiotics and the emergence of resistance ( Bassetti et al., 2022).

Second, predictive models can enhance the national surveillance systems by identifying the emerging resistance trends and forecasting future resistance patterns. Such insights could inform resource allocation, design of AMS programs, and infection prevention strategies.

Third, these models may support pharmaceutical research and development by identifying geographic regions where resistance to specific antibiotics is increasing, thereby guiding clinical trial site selection and prioritization of novel antimicrobial agents.

From a health-economic perspective, predictive AMR models may also contribute to cost-effective healthcare delivery. The earlier identification of likely effective therapies could reduce unnecessary antibiotic use, shorten hospital stays, and decrease the incidence of treatment failures requiring costly second-line therapies. Integrating predictive analytics into stewardship programs may therefore yield both clinical and economic benefits.

At the policy level, the integration of machine learning-based predictive tools into national AMR surveillance frameworks could strengthen evidence-based decision-making. Governments and global health organizations are increasingly emphasizing data-driven approaches to combat AMR. The development of scalable predictive models based on existing surveillance infrastructure represents a practical step towards operationalizing this vision.

## Limitations

Several limitations should be considered when interpreting the findings of this study.

First, the dataset is derived from surveillance systems rather than population-based sampling, which may introduce selection bias towards tertiary care centers and urban healthcare facilities.

Second, class imbalance within susceptibility categories may inflate apparent performance metrics, particularly when resistant isolates represent a minority of observations.

Third, the dataset lacks detailed patient-level clinical information such as prior antibiotic exposure, comorbidities, hospitalization history, and treatment outcomes. The absence of these variables limits the ability of the models to generate individualized predictions.

Finally, temporal drift in resistance patterns was not explicitly modeled in the current analysis. Resistance prevalence can change rapidly over time, and models trained on historical data may require periodic retraining to maintain predictive accuracy.

Future research should therefore focus on prospective validation of predictive models within clinical workflows, integration of temporal modeling approaches, and incorporation of additional clinical variables that may improve predictive accuracy.

## Conclusion

AMR continues to threaten the effectiveness of modern antimicrobial therapy and represents a growing challenge for global health systems. Strengthening surveillance capacity and improving the timeliness of susceptibility information are therefore central to AMS and effective clinical decision-making. In this study, we explored the potential of leveraging global antimicrobial surveillance data to develop interpretable machine learning models capable of predicting antimicrobial susceptibility outcomes.

By integrating a rule-based MIC interpretation tool with antibiotic-specific predictive modeling, our approach demonstrates how routinely collected surveillance data can be transformed into analytical tools that extend beyond traditional descriptive reporting. The results suggest that epidemiological and microbiological metadata contain meaningful predictive signals that can support probabilistic estimations of resistance patterns. Importantly, the use of explainable machine learning methods enabled the identification of key features influencing model predictions, enhancing transparency and interpretability.

The findings also highlight the potential value of surveillance-driven predictive modeling within AMS frameworks. In settings where laboratory turnaround times remain prolonged or diagnostic infrastructure is limited, predictive insights derived from existing surveillance datasets may help support empirical prescribing decisions and guide stewardship interventions. Such approaches may be particularly relevant to LMICs where the burden of resistant infections is high but access to advanced genomic diagnostics remains limited.

Beyond immediate clinical applications, the integration of predictive analytics with large-scale surveillance platforms may also contribute to improved resistance monitoring and policy planning. By identifying emerging resistance patterns and geographic heterogeneity in susceptibility trends, predictive models can support more responsive stewardship strategies and inform resource allocation for AMR control.

Several challenges, however, remain. Including the need for external validation across independent surveillance datasets, incorporation of patient-level clinical variables, and exploration of temporal forecasting methods capable of capturing evolving resistance dynamics. Future work should also examine the integration of surveillance-based prediction tools within clinical decision support systems and national AMS programs.

In conclusion, this study demonstrates that ML approaches applied to global antimicrobial surveillance datasets can generate interpretable predictions of antimicrobial susceptibility using routinely collected epidemiological data. This study illustrates how surveillance data may be leveraged to enhance AMS, strengthen resistance monitoring, and support evidence-based responses to the threat of AMR.

## Mathematical scripts

No explicit mathematical equations were used. All modeling was implemented using standard machine learning algorithms (XGBoost) in Python.

## Ethics and consent

Ethical approval and consent were not required. Ethical approval was not required for this study, as it used secondary, de-identified data from the Pfizer ATLAS database.

## Data Availability

The antimicrobial susceptibility data used in this study were obtained from the Pfizer ATLAS program. Access to this dataset is available through the Vivli data sharing platform, subject to data access agreements, using the following information: Data contributor name: Pfizer (AMR); DOI: https://doi.org/10.25934/PR00007930; AMR ID: VIV00007930; and Dataset ID: ATLAS_Antibiotics. The interpretive model was trained using MIC breakpoints and guidelines in the 35th edition of the CLSI 2025 standards ( Clinical and Laboratory Standards Institute, 2025). No new primary data were generated in this study. No extended data are associated with this article.

## Software availability

Source code available from:

https://github.com/davidgichohi/amr-predictor

https://github.com/davidgichohi/AMR-MIC-1

Archived software available from:

https://doi.org/10.5281/zenodo.19681458

https://doi.org/10.5281/zenodo.19681397

License:

MIT License.

Software environment: Analysis and modeling were done using Python 3.10, XGBoost, and SHAP.
