# Surveillance-Driven Machine Learning for Prediction of Antimicrobial Susceptibility: An Explainable Modeling Framework using the Pfizer ATLAS Dataset (2004 - 2023)

**DOI:** 10.12688/wellcomeopenres.26477.2

**Published:** 2026-09-21

**Authors:** Raphael Mutua, David Gichohi, Samuel K. Ndegwa, Rahma O. Golicha, Frida Njeru, Benson Kituku

**Affiliations:** 1 Center for Microbiology Research, Kenya Medical Research Institute, Nairobi, Nairobi County, 54840-00200, Kenya; 2 Department of Computer Science, Dedan Kimathi University of Technology, Nyeri, Nyeri County, 657-10100, Kenya

**Keywords:** Antimicrobial Resistance, Machine Learning, AMR Surveillance, Predictive Modelling, Antimicrobial Susceptibility

## Abstract

**Background:**

Antimicrobial resistance (AMR) is a growing global public health threat, particularly in low- and middle-income countries (LMICs), where delayed antimicrobial susceptibility testing (AST) and limited diagnostic capacity complicate timely treatment and antimicrobial stewardship (AMS). Although large-scale surveillance programmes routinely collect antimicrobial susceptibility data, these datasets remain underutilised for predictive analytics.

**Methods:**

We developed a surveillance-driven machine learning (ML) framework using isolate-level data from African sites participating in the Pfizer Antimicrobial Testing Leadership and Surveillance (ATLAS) programme between 2019 and 2023. Seven antibiotic-specific Extreme Gradient Boosting (XGBoost) models were developed to predict antimicrobial susceptibility using routinely collected microbiological, demographic, clinical, and geographical metadata. Model development included structured data preprocessing, RandomOverSampler-based class balancing restricted to the training data, hyperparameter optimisation using stratified cross-validation, and evaluation on a held-out test dataset. A rule-based MIC interpretation system and interactive dashboard were developed to demonstrate implementation of the analytical workflow.

**Results:**

The antibiotic-specific models demonstrated moderate predictive performance, with test accuracies ranging from 57% to 76%. Ceftazidime-Avibactam achieved the highest test accuracy (76%), followed by Gentamicin (65%) and Imipenem (64%), while Amikacin showed the lowest performance (57%). Feature-importance analysis identified bacterial species as consistently among the most influential predictors. In contrast, bacterial family, country of isolate collection, specimen source, clinical specialty, and demographic characteristics contributed to varying degrees across antibiotics. Performance was generally stronger for the more frequently represented susceptible class than for intermediate and resistant isolates.

**Conclusion:**

Routinely collected AMR surveillance data can support antibiotic-specific ML predictions without requiring genomic sequencing or detailed patient-level clinical information. This study provides a proof-of-concept framework for surveillance-driven predictive analytics that could complement conventional AMR surveillance and AMS. External validation, calibration, prospective clinical evaluation, and implementation studies are required before routine deployment.

## Plain Language summary

Antimicrobial resistance (AMR) occurs when bacteria no longer respond to medicines used to treat infections, making illnesses harder to treat and increasing the risk of severe outcomes. This is a growing global health problem, particularly in Low- and Middle-Income Countries (LMICs) where access to rapid diagnostic testing and Antimicrobial Stewardship (AMS) programs may be limited.

In many healthcare settings, laboratory testing to determine which antibiotics are effective can take 48–72 hours. During this time, patients are often treated with broad-spectrum antibiotics without knowing whether they will work. This can lead to unnecessary antibiotic use, higher treatment costs, and the development of further resistance.

This study explored whether routinely collected AMR surveillance data can be used to predict antibiotic effectiveness. Using data from the Pfizer ATLAS surveillance program, we developed computer-based models that estimate the likelihood that a specific antibiotic will be effective against a bacterial infection. These predictions are based on factors such as the type of bacteria, geographic location, and sample source.

We also developed a rule-based tool that interprets laboratory measurements into standard susceptibility categories and an interactive dashboard that allows users to explore resistance trends and model predictions in a user-friendly way.

The results show that surveillance data can be used to generate meaningful predictions about antibiotic effectiveness, even without advanced laboratory or genomic data. These tools could support earlier and more informed treatment decisions, improve antibiotic use, and strengthen monitoring of resistance patterns.

However, the accuracy of these predictions depends on the quality and completeness of available data, and further validation is needed before routine clinical use.

Overall, this study demonstrates how existing surveillance data can be transformed into practical tools to support better use of antibiotics and help slow the spread of antimicrobial resistance, particularly in resource-limited settings.

## Introduction

Antimicrobial resistance (AMR) remains one of the most pressing global public health threats, compromising the effective treatment of bacterial infections and increasing morbidity, mortality, healthcare costs, and the risk of treatment failure worldwide ( Baekkeskov & Pierre, 2024). Recent estimates indicate that bacterial AMR was directly responsible for approximately 1.27 million deaths and associated with 4.95 million deaths globally in 2019, with low- and middle-income countries (LMICs) bearing a disproportionate share of the burden owing to limited diagnostic capacity, constrained antimicrobial stewardship (AMS) programmes, and delayed access to effective treatment ( Murray et al., 2022; Kariuki et al., 2022). These findings underscore the urgent need for improved surveillance systems, timely diagnostics, and innovative stewardship strategies capable of mitigating the emergence and spread of resistant pathogens ( Ahmed, 2025).

Accurate antimicrobial susceptibility testing (AST) remains the cornerstone of evidence-based antimicrobial therapy. However, conventional phenotypic AST methods typically require 48-72 hours after specimen collection before susceptibility results become available, during which clinicians frequently initiate empirical broad-spectrum antimicrobial therapy ( Syal et al., 2017). While this approach is often clinically necessary, inappropriate empirical prescribing contributes to selection pressure that accelerates the emergence and dissemination of AMR. These challenges are particularly pronounced in many LMICs, where laboratory infrastructure, trained personnel, and access to rapid diagnostic technologies remain limited, resulting in greater reliance on syndromic management and empirical antibiotic use ( Reali et al., 2025). Delayed susceptibility results also have important economic implications, including increased drug expenditure, prolonged hospitalisation, and higher risks of treatment failure, thereby imposing substantial costs on already constrained healthcare systems ( Lucidi et al., 2017; Sulis et al., 2022).

Global surveillance platforms have been established to monitor emerging resistance patterns and inform public health responses. Programmes such as the World Health Organization Global Antimicrobial Resistance and Use Surveillance System (GLASS) and the Pfizer Antimicrobial Testing Leadership and Surveillance (ATLAS) programme compile standardized AST data from participating laboratories across multiple countries, providing valuable epidemiological insights into the geographic and temporal distribution of resistance ( Leclerc et al., 2020; WHO, 2023; Jia et al., 2023). Although these surveillance datasets have traditionally been used for descriptive epidemiological reporting, their scale, longitudinal nature, and standardized microbiological data present an opportunity to support predictive modelling approaches that could complement existing surveillance activities.

Recent advances in ML have generated considerable interest in developing predictive models capable of estimating antimicrobial susceptibility before conventional laboratory results become available. Most published studies have relied on electronic health records, previous microbiology results, laboratory biomarkers, patient clinical characteristics, or whole-genome sequencing data to predict resistant phenotypes ( Kim et al., 2022; Ren et al., 2022). Systematic reviews have consistently demonstrated that ensemble learning algorithms, including Random Forest and gradient boosting methods such as XGBoost, frequently achieve strong predictive performance across a range of bacterial pathogens and antimicrobial agents. However, these reviews also highlight several persistent methodological limitations, including inadequate reporting of model development, inconsistent validation strategies, limited external validation, heterogeneous preprocessing methods, and uncertainty regarding model generalisability across healthcare settings ( Lv & Wang, 2024; Ardila et al., 2025).

Prediction models based on genomic data have demonstrated particularly high accuracy because they directly identify genetic determinants of AMR ( Kim et al., 2022; Ren et al., 2022). Nevertheless, these approaches require sequencing infrastructure, specialised bioinformatics expertise, and substantial computational resources that remain unavailable in many routine diagnostic laboratories, particularly within resource-constrained settings ( Olorunkosebi et al., 2025; Nkhono et al., 2026). Similarly, models developed using electronic health records often depend on detailed patient-level clinical information, previous microbiological cultures, or institutional prescribing histories that may not be routinely available within national AMR surveillance programmes. Consequently, many existing prediction models rely on data sources that are difficult to implement within the surveillance systems available in many LMICs.

An emerging alternative is the use of routinely collected surveillance data to support antimicrobial susceptibility prediction. International surveillance programmes, such as the Pfizer ATLAS database, provide standardized microbiological and epidemiological information collected across multiple countries over extended periods, creating opportunities to develop predictive models using surveillance metadata already generated during routine laboratory practice ( Leclerc et al., 2020).

Recent work has demonstrated the feasibility of applying ML to surveillance datasets to predict antimicrobial susceptibility. However, many studies have analysed global datasets, focused primarily on overall predictive performance, or have neither specifically evaluated implementation in resource-limited settings nor emphasized model interpretability ( Valavarasu et al., 2025).

For example, Valavarasu et al. (2025) developed ML models using the global Pfizer ATLAS dataset and demonstrated excellent predictive performance across multiple bacterial pathogens. However, their work primarily emphasized algorithm performance and feature importance rather than evaluating implementation within LMIC-focused surveillance systems, standardizing MIC interpretation workflows, or providing an integrated analytical framework that combines standardized MIC interpretation, interpretable ML, and interactive visualization. More broadly, previous surveillance-based prediction studies have generally relied on datasets from high-income settings or have not examined whether routinely collected surveillance metadata alone, without patient-level clinical information or genomic sequencing, can provide sufficiently informative predictors of antimicrobial susceptibility across WHO priority bacterial pathogens ( Yelin et al., 2019; Feretzakis et al., 2021).

The present study was designed to address these methodological and implementation gaps. Rather than developing a universally applicable global prediction model, we intentionally restricted our analyses to African surveillance data as a proof of concept for settings where delayed AST turnaround times, limited laboratory infrastructure, and restricted access to advanced molecular diagnostics continue to constrain evidence-based antimicrobial prescribing. We further focused on bacterial species included in the WHO bacterial priority pathogens list because these organisms represent the greatest public health concern and remain priorities for global AMR surveillance, AMS, and research investment ( WHO, 2024).

Unlike previous surveillance-based studies, this work integrates several complementary components within a single transparent analytical framework. We developed antibiotic-specific ML models using routinely collected surveillance metadata from the Pfizer ATLAS database. We implemented a standardized rule-based workflow for interpreting minimum inhibitory concentration (MIC) values using the susceptibility interpretations provided in the ATLAS dataset, optimised the antibiotic-specific models, and identified the epidemiological variables most strongly associated with antimicrobial susceptibility. Finally, we developed a prototype dashboard to visualise model outputs. Together, these components provide a reproducible framework for antimicrobial susceptibility prediction and surveillance. By combining interpretive algorithms with explainable ML tools, this study demonstrates how routinely collected surveillance data can be transformed into predictive tools that may complement AMS and surveillance activities, particularly in resource-limited settings.

## Methods

### Study design and data source

This study was designed as a retrospective secondary analysis of global AMR surveillance data derived from the Pfizer ATLAS program.

The ATLAS database is one of the largest publicly accessible global AMR surveillance repositories, containing microbiological susceptibility data collected between 2004 and 2023 from participating healthcare institutions across more than 80 countries. The database includes MIC measurements together with organism characteristics, specimen information, geographical location, patient demographics, and AST results determined using internationally accepted laboratory standards.

In the dataset, AST interpretations were provided according to Clinical and Laboratory Standards Institute (CLSI) breakpoints. Although the ATLAS platform also supports interpretation using European Committee on Antimicrobial Susceptibility Testing (EUCAST) breakpoints, all analyses in this study were performed using the CLSI-derived susceptibility interpretations supplied within the dataset. Consequently, no breakpoint harmonization or recalculation was required during model development. CLSI and EUCAST interpretations were therefore not combined during model development, and the breakpoint system was not included as a predictor variable. Restriction of the predictive analysis to African isolates collected between 2019 and 2023 also reduced the period over which temporal changes in breakpoint definitions and laboratory practices could influence the modelling dataset. This restriction was intended to improve the geographical relevance of the prediction models while ensuring that the training data reflected contemporary AMR patterns. Predictor variables included bacterial species, bacterial family, country of origin, patient age group, gender, medical specialty, and specimen source, representing information that is routinely available at or shortly after specimen collection. Only bacterial species represented within the WHO Bacterial Priority Pathogens framework were retained for the analytical population because of their relevance to AMR surveillance and public health. Each isolate constituted an independent observation. Analyses were conducted using de-identified secondary surveillance data.

The study comprised two complementary components. The first component developed a rule-based antimicrobial susceptibility interpretation engine that automatically converts MIC values into categorical susceptibility interpretations (Susceptible, Intermediate, or Resistant) using standardized clinical breakpoint rules. The second component developed supervised ML models capable of predicting antibiotic susceptibility from routinely available demographic and microbiological characteristics before laboratory susceptibility testing results become available. Together, these components demonstrate how surveillance data can be transformed into practical decision-support tools for AMS, surveillance, and clinical decision-making.

Rather than developing a single multiclass prediction model for all antimicrobial agents, separate antibiotic-specific models were constructed. This approach allowed each model to learn resistance patterns unique to an individual antimicrobial while reducing the complexity associated with heterogeneous susceptibility distributions across antibiotics. Antibiotics selected for model development were those with sufficient numbers of susceptible observations to support reliable model training and evaluation. The implemented prediction system included models for Gentamicin, Ceftazidime-Avibactam, Amikacin, Imipenem, Piperacillin-Tazobactam, Ceftolozane-Tazobactam, and Ceftazidime.

### Data preparation

Before model development, the Pfizer ATLAS dataset underwent a structured preprocessing workflow to ensure data quality, consistency, and suitability for ML analysis. For each antibiotic-specific analysis, isolates lacking the target susceptibility result or any of the required predictor variables were excluded using complete-case analysis. Preprocessing was therefore performed independently for each antibiotic-specific dataset, ensuring that only observations with complete predictor information and a valid susceptibility interpretation were retained for model development.

The prediction models were developed using routinely available demographic and microbiological information that could realistically be obtained before AST results become available. The predictor variables used were selected because of their documented association with AMR patterns and their availability within the surveillance dataset. The outcome variable for each model was the categorical antimicrobial susceptibility result corresponding to a specific antibiotic. Categorical predictor variables were transformed into numerical representations suitable for ML algorithms. Specifically, bacterial species, bacterial family, country of origin, patient age group, gender, medical specialty, and specimen source were transformed using one-hot encoding. Susceptibility outcomes were label-encoded as Resistant = 0, Intermediate = 1, and Susceptible = 2 for model fitting. These numerical class labels were used solely for computational purposes and did not imply an ordinal relationship between the susceptibility categories. No feature scaling or standardisation was applied because the final prediction models were tree-based classifiers.

### Development of an interactive dashboard

We developed an integrated, web-based interactive dashboard for the analysis, model output, and visualization of AMR resistance trends ( Figure 1). The dashboard enabled users to:

1. Explore AMR trends across countries, organisms and antibiotics
2. Interact with an interpretive model that translates MIC values into clinical susceptibility categories based on organism, specimen source, and antibiotic class ( Figure 2).
3. Engage with a predictive model to identify the most effective antibiotic based on patient and isolate’s characteristics, including species, family, country, gender, age group, clinical specialty, and specimen source ( Figure 3).

**Figure 1.**
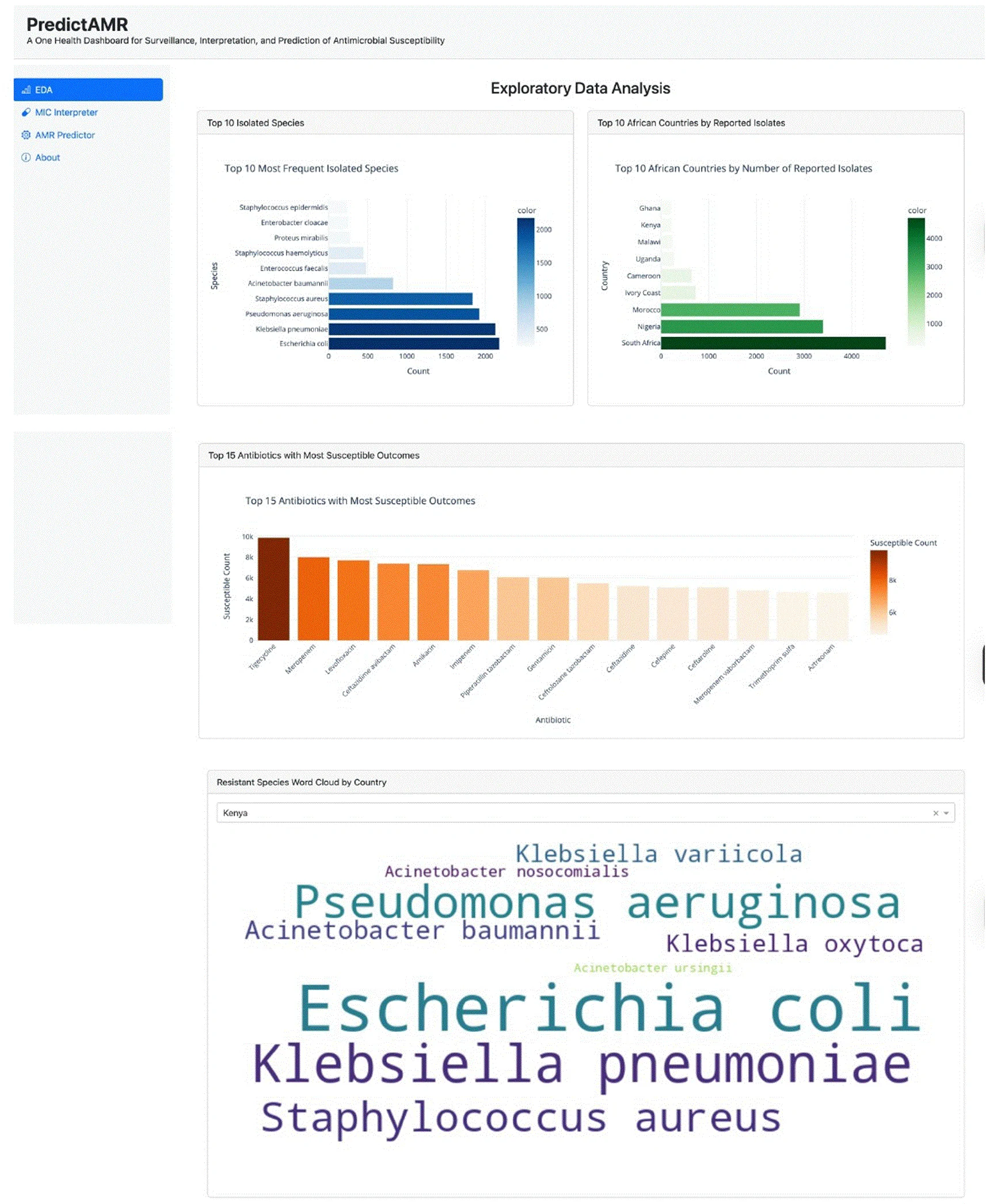
Interactive dashboard for AMR surveillance and prediction. The interface allows users to explore AMR trends across countries, organisms, and antibiotics through dynamic filtering and visualization tools.

**Figure 2.**
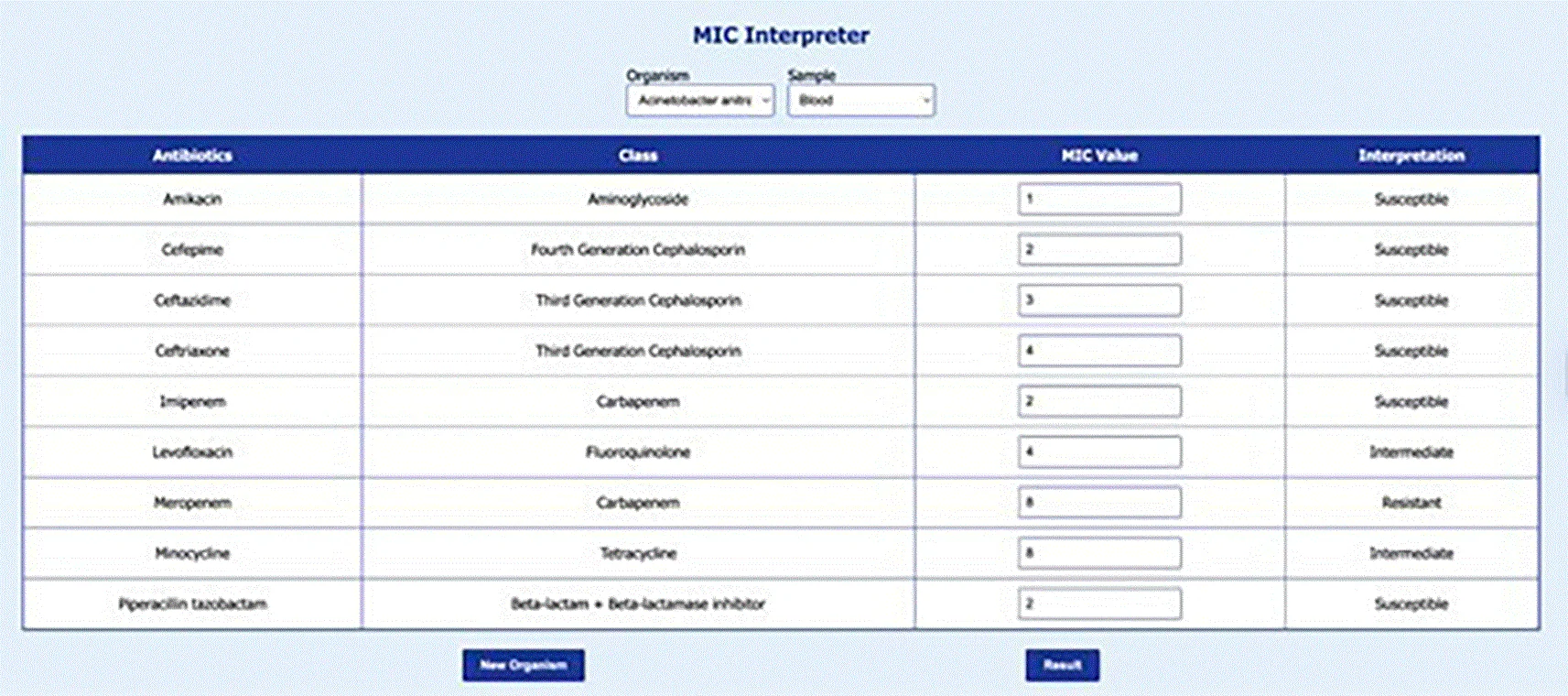
Rule-based MIC interpretive tool for susceptibility classification. The tool converts MIC values into standardized susceptibility categories based on organism, specimen source, and antibiotic class.

**Figure 3.**
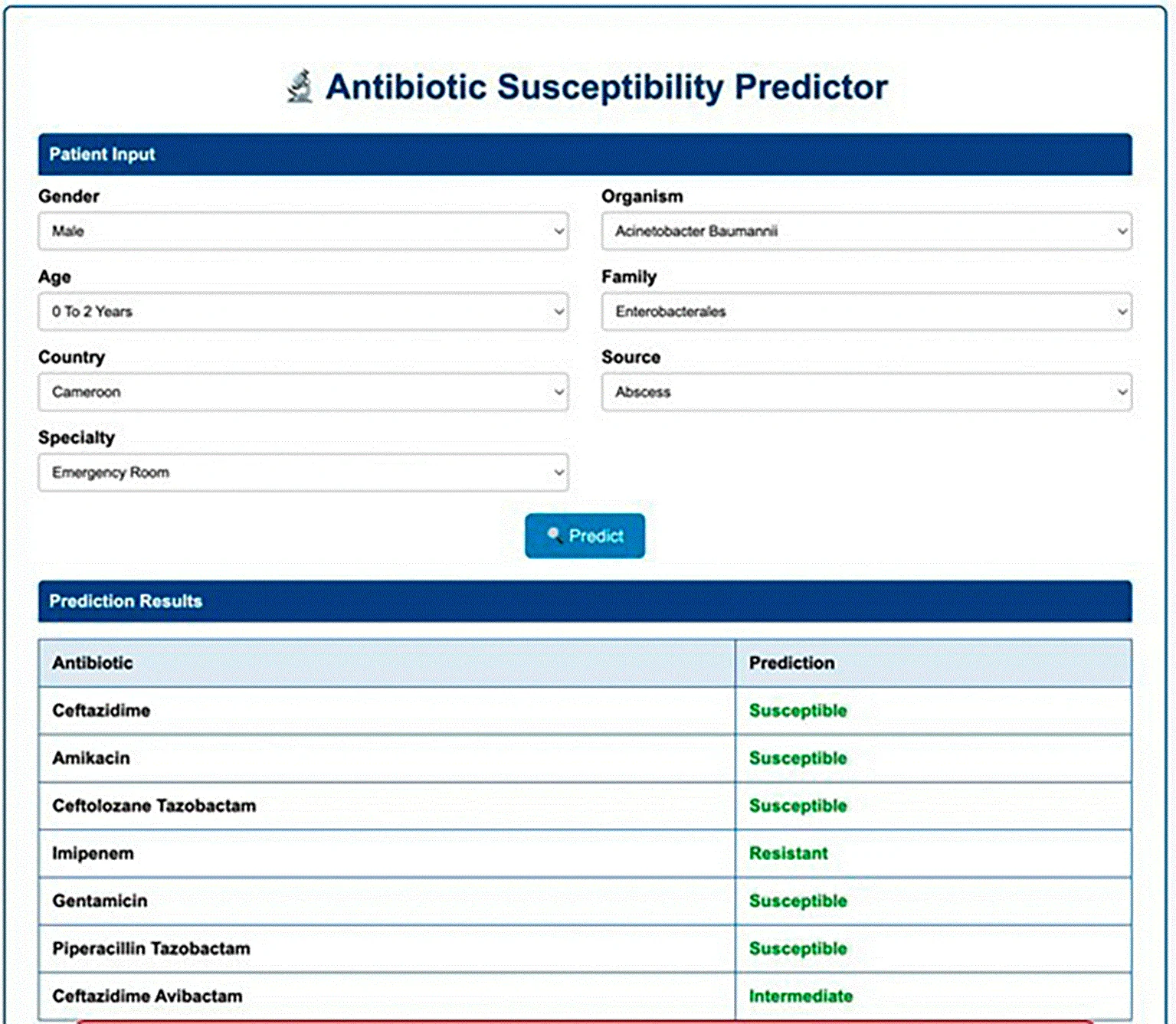
Machine learning model for antimicrobial susceptibility prediction. The model generates probabilistic predictions of antibiotic effectiveness based on epidemiological and clinical input variables.

The dashboard served as a central interface for surveillance-driven decision-making, enabling clinicians, researchers, and policymakers to derive actionable insights from complex AMR datasets.

### Training predictive machine learning models

Seven independent supervised ML models were developed to predict antimicrobial susceptibility for the selected antibiotics. Rather than constructing a single multi-output classifier, a separate model was trained for each antimicrobial agent to capture antibiotic-specific resistance patterns and improve model interpretability. This approach recognizes that resistance mechanisms, prevalence, and susceptibility distributions differ substantially between antimicrobial agents and therefore require individualized modelling strategies. The dataset for each antibiotic was split into training and testing subsets using stratified sampling to preserve the distribution of susceptibility classes across both datasets. Model development was performed exclusively on the training dataset, while the testing dataset was reserved for evaluation after model optimisation. Specifically, 80% of observations were allocated to the training dataset and 20% to the held-out testing dataset using a stratified train-test split. Because several antibiotic-specific datasets exhibited class imbalance, RandomOverSampler (random_state = 42) was applied exclusively to the training dataset to increase representation of minority susceptibility classes. The held-out test dataset was not oversampled and was not used during model fitting or hyperparameter optimisation, thereby reducing the risk of information leakage and preserving an independent dataset for final evaluation. Extreme Gradient Boosting (XGBoost) algorithm was selected as the prediction model based on its ability to model complex nonlinear relationships, robustness to heterogeneous clinical data, computational efficiency, and strong predictive performance in structured tabular datasets.

### Hyperparameter tuning

Model hyperparameters were optimised using an automated grid search strategy implemented within the training dataset. Candidate combinations of XGBoost hyperparameters were systematically evaluated using stratified cross-validation. The optimisation process explored combinations of key model parameters, including the number of boosting iterations (n_estimators), maximum tree depth (max_depth), learning rate (learning_rate), and subsampling proportion (subsample). The parameter grid evaluated n_estimators values of 50 and 100, max_depth values of 3, 5, and 7, learning_rate values of 0.05 and 0.10, and subsample values of 0.8 and 1.0. Hyperparameter optimisation was performed using GridSearchCV with three-fold stratified cross-validation, with the weighted F1-score used as the optimisation criterion to account for unequal susceptibility-class frequencies. The optimal parameter combination identified through cross-validation was subsequently used to train the final prediction model for each antibiotic. All hyperparameter optimisation was conducted exclusively within the training dataset. The held-out testing dataset was not used during model selection or optimisation and was reserved for final evaluation of the optimised models.

### Model evaluation

Performance evaluation included overall classification accuracy together with precision, recall, and F1-score derived from confusion matrices. Because susceptibility classes were imbalanced, weighted precision, weighted recall, and weighted F1-score were considered alongside overall accuracy, and class-specific performance was examined for Susceptible, Intermediate, and Resistant isolates. Confusion matrices were used to examine the distribution of correct classifications and misclassifications across susceptibility categories. Receiver operating characteristic curves and the area under the ROC curve (AUROC) were not included in the final evaluation. Formal model calibration, confidence intervals for performance estimates, and statistical hypothesis tests comparing the antibiotic-specific classifiers were also not undertaken. Because each classifier represented a different antibiotic-specific prediction task with different sample sizes and susceptibility distributions, comparisons between models were descriptive. External validation using an independent surveillance dataset was not performed because no suitable independent dataset with comparable variables, antibiotic susceptibility information, and outcome definitions was available for this analysis. Feature importance was extracted from each trained XGBoost model to identify the variables contributing most strongly to antimicrobial susceptibility prediction. The important features help to interpret model behaviour and to assess the relative influence of demographic, microbiological, and geographical characteristics on resistance prediction. Feature-importance measures were interpreted as indicators of predictive contribution rather than evidence of causal relationships with AMR.

### Rule-based MIC interpretation tool

In parallel with the predictive modelling framework, a rule-based antimicrobial susceptibility interpretation system was developed to automate interpretation of MIC values. The interpretation engine converts experimentally determined MIC values into categorical susceptibility results. Clinical breakpoint rules were derived from the surveillance data and encoded into structured JavaScript Object Notation (JSON) configuration files. The rule engine maps bacterial organisms, specimen sources, antimicrobial agents, and corresponding MIC thresholds to their respective susceptibility categories (S/I/R). At deployment, users provide organism information together with observed MIC values, after which the application interprets and retrieves the appropriate breakpoint definitions and generates standardized susceptibility interpretations.

Separating the interpretation engine from the predictive modelling framework enables two complementary clinical workflows. The rule-based component standardises interpretation of laboratory-derived MIC measurements, whereas the predictive models estimate likely susceptibility before antimicrobial susceptibility testing results become available. Together, these components provide decision support across different stages of the clinical microbiology workflow. The rule-based interpretation system was not subjected to a separate prospective or clinical validation study and should therefore be regarded as an implementation of encoded breakpoint rules rather than an independently validated diagnostic system.

### Software implementation and reproducibility

All data preprocessing, exploratory data analysis, model development, evaluation, and deployment were implemented using Python with open-source libraries. These included Pandas for data manipulation, NumPy for numerical computation, Scikit-learn for data preprocessing and model evaluation, XGBoost for predictive modelling, and plotly for interactive data visualisation. Web-based deployment of the predictive models and antimicrobial susceptibility interpretation system was implemented using Python-based application frameworks, enabling translation of the developed models into interactive decision-support tools. The analytical workflow was developed using Python 3.10 in Google Colab environments. The modelling workflow additionally used imbalanced-learn for RandomOverSampler-based class balancing and GridSearchCV within Scikit-learn for model optimisation.

To facilitate reproducibility and transparency, the complete source code for the project has been made publicly available through version-controlled GitHub repositories. Separate repositories were maintained for the rule-based MIC interpretation engine, the ML prediction models, and the integrated AMR dashboard. These repositories contain the implementation code, application logic, deployment configuration, and supporting documentation required to reproduce the computational workflow. The predictive modelling repository ( https://github.com/davidgichohi/amr-predictor) contains the complete ML pipeline, including data preprocessing routines, feature engineering procedures, model training scripts, hyperparameter optimisation workflow, model serialization, and application code used during deployment. The MIC interpretation repository ( https://github.com/davidgichohi/AMR-MIC-1) contains the rule engine together with the structured breakpoint definitions used to translate MIC values into categorical antimicrobial susceptibility interpretations, JSON rule files, software dependency specification, and Dockerfile used for deployment. The dashboard repository ( https://github.com/davidgichohi/PredictAMR) integrates both components into a unified web-based platform for visualization, exploration of surveillance data, and prediction of antimicrobial susceptibility. These resources would facilitate future extension of the framework by other researchers.

## Results

### Exploratory data analysis

The filtered analytical dataset comprised 13,343 isolate-level records from nine African countries. The most frequently represented bacterial species were *Escherichia coli* (2,179 isolates), *Klebsiella pneumoniae* (2,129), *Pseudomonas aeruginosa* (1,922), *Staphylococcus aureus* (1,837), and *Acinetobacter baumannii* (822). Enterobacteriaceae constituted the largest bacterial family (4,123 isolates). South Africa, Nigeria, and Morocco contributed the largest numbers of isolates, while blood, wound, and urine were the most frequently represented specimen sources.

Exploratory analysis of the Pfizer ATLAS dataset identified substantial variation in antimicrobial susceptibility across bacterial species, geographical regions, specimen sources, and antimicrobial agents. The dataset demonstrated considerable heterogeneity in susceptibility distributions, with several antibiotics exhibiting pronounced class imbalance characterized by a predominance of susceptible isolates and relatively fewer intermediate and resistant observations. For example, amikacin included 7,339 susceptible, 1,309 resistant, and 589 intermediate observations, whereas ceftazidime included 5,211 susceptible, 3,784 resistant, and 357 intermediate observations.

Descriptive analyses further identified notable differences in resistance profiles among bacterial species and participating countries. These exploratory findings informed antibiotic selection for predictive modelling by identifying antimicrobial agents with sufficient observations to support robust model development.

### Rule-based MIC interpretation tool

A rule-based antimicrobial susceptibility interpretation system was developed to automate interpretation of MIC values. The system applies organism-specific breakpoint rules encoded in structured JSON files to classify laboratory-derived MIC values as S, I, or R. The web-based implementation enables users to select the bacterial organism and specimen source, enter MIC values for the relevant antimicrobial agents, and receive standardized susceptibility interpretations in real time. The tool proved effective in providing a consistent and automated approach to MIC interpretation across the supported organism-antimicrobial combinations.

### Performance of XGBoost prediction models

Seven antibiotic-specific XGBoost prediction models were developed and evaluated independently. Each model was trained using demographic and microbiological characteristics routinely available before AST results become available. Model performance varied across antimicrobial agents, reflecting differences in resistance prevalence and class distribution within each dataset. Training accuracies ranged from approximately 73% to 86%, whereas testing accuracies ranged between 57% and 76%, indicating moderate generalization performance across the evaluated antibiotics. The training and testing accuracies of the models are shown in Table 1. The observed reduction in testing accuracy relative to training accuracy suggests that model performance was influenced by the inherent class imbalance present within several AST datasets rather than by overfitting alone. Among the evaluated models, the Ceftazidime-Avibactam classifier demonstrated the highest testing performance, whereas models developed for Amikacin, Piperacillin-Tazobactam, and Ceftazidime exhibited comparatively lower predictive accuracy. These differences likely reflect variability in susceptibility distributions and resistance epidemiology across individual antibiotics.

**Table 1.** Predictive performance of the developed models.

Model	Training accuracy	Testing accuracy
Ceftazidime avibactam	86%	76%
Amikacin	76%	57%
Imipenem	78%	64%
Piperacillin tazobactam	73%	62%
Gentamicin	79%	65%
Ceftolozane tazobactam	83%	62%
Ceftazidime	74%	62%

Classification reports additionally demonstrated variation in precision, recall, and F1-score across susceptibility classes. Performance was generally stronger for the more frequently represented susceptible class than for the intermediate and resistant classes, consistent with the class imbalance identified during exploratory analysis. Confusion matrices similarly demonstrated that misclassification occurred disproportionately among the less frequently represented susceptibility categories. Accordingly, overall accuracy should be interpreted together with class-specific precision, recall, and F1-score rather than as a stand-alone measure of model performance.

### Feature importance and model interpretation

Feature importance analysis demonstrated that bacterial species was consistently among the most influential predictors across the developed XGBoost models, highlighting the strong association between organism identity and antimicrobial susceptibility. Geographical location, specimen source, and patient demographic characteristics also contributed to prediction, although their relative importance varied among antibiotics. These findings indicate that routinely collected clinical and microbiological information contains meaningful predictive signals that can support empirical antimicrobial decision-making before laboratory susceptibility testing is completed.

## Discussion

### Principal findings

This study developed and evaluated antibiotic-specific XGBoost models for predicting antimicrobial susceptibility using routinely collected surveillance data from the Pfizer ATLAS programme. Across seven clinically important antibiotics, the models achieved moderate predictive performance using only routinely collected microbiological and epidemiological variables, demonstrating the feasibility of surveillance-driven ML without requiring genomic sequencing or detailed patient-level clinical information. Unlike many previous ML studies, our framework was intentionally designed around variables routinely collected within existing AMR surveillance systems. This design improves the potential applicability of the approach in LMICs, where access to genomic sequencing, advanced diagnostics, and comprehensive electronic health records remains limited.

An additional contribution of this study is the integration of standardized MIC interpretation, data preparation, antibiotic-specific XGBoost models, performance evaluation, and interactive visualization within a single reproducible analytical workflow. Rather than presenting these components as independent tools, the framework illustrates how routinely collected surveillance data can be transformed into a practical foundation for surveillance-driven predictive analytics. Importantly, the proposed framework should be regarded as a proof of concept demonstrating technical feasibility rather than a clinically validated decision-support system. Prospective clinical evaluation, external validation, and implementation studies remain necessary before routine deployment.

### Comparison with existing literature

ML approaches for predicting AMR have expanded rapidly over the past decade, with most published models relying on genomic sequencing, electronic health records, or previous microbiological results ( Kim et al., 2022; Ren et al., 2022). Although these approaches frequently report excellent predictive performance, they often require data sources and infrastructure that remain unavailable in many routine clinical laboratories, particularly within LMICs ( Olorunkosebi et al., 2025).

More recently, several studies have explored the use of routinely collected surveillance data as an alternative source of predictive information ( Chindelevitch et al., 2022; Valavarasu et al., 2025). Consistent with these reports, our findings demonstrate that routinely collected surveillance metadata contain sufficient information to support antimicrobial susceptibility prediction. Furthermore, our study extends previous work by focusing exclusively on WHO bacterial priority pathogens from African surveillance data, thereby providing a regional proof of concept relevant to LMIC settings. The study also integrates standardized MIC interpretation, data preparation, model development, performance evaluation, and interactive visualization within a single reproducible workflow, illustrating how predictive analytics can be incorporated into existing surveillance systems. The observed variation in predictive performance across antibiotics is also consistent with previous studies showing that model performance is influenced not only by algorithm selection but also by the epidemiology of resistance, class imbalance, predictor availability, and the biological complexity of resistance mechanisms ( Lv & Wang, 2024; Ardila et al., 2025).

### Interpretation of model performance

Feature importance analysis indicated that bacterial species was consistently among the most influential predictors of antimicrobial susceptibility across the developed models, while bacterial family, country of isolate collection, specimen source, clinical specialty, and demographic characteristics contributed to varying degrees across antibiotics. These findings are biologically plausible because intrinsic resistance mechanisms differ substantially between bacterial taxa, while geographic variation reflects differences in antimicrobial consumption, infection prevention practices, healthcare infrastructure, diagnostic capacity, and circulating resistance determinants ( Coque et al., 2023; Hossain & Chowdhury, 2024; Ye et al., 2025).

Specimen source and clinical specialty also contributed to prediction performance, suggesting that the clinical context provides additional predictive information beyond organism identity alone. The relative contribution of individual predictors varied between antibiotic-specific models, indicating that the predictive information captured by microbiological, epidemiological, and demographic characteristics differed according to the antimicrobial agent being modelled.

Predictive performance varied across antibiotics, reflecting differences in both the underlying epidemiology of resistance and the characteristics of the available surveillance data. Ceftazidime-avibactam demonstrated the highest test accuracy (76%), followed by gentamicin (65%) and imipenem (64%), whereas amikacin showed the lowest test accuracy (57%). The remaining models achieved test accuracies of approximately 62%. Differences in performance across antibiotics may reflect variation in susceptibility-class distributions, sample characteristics, and the extent to which routinely collected surveillance variables capture antibiotic-specific resistance patterns.

Across the antibiotic-specific models, prediction performance was generally stronger for the more frequently represented susceptible class than for intermediate and resistant isolates. Confusion-matrix evaluation similarly indicated that misclassification occurred disproportionately among the less frequently represented susceptibility categories, demonstrating that minority-class prediction remained challenging despite the use of RandomOverSampler. Collectively, these findings demonstrate that routinely collected surveillance data contain meaningful predictive information and provide a practical foundation for surveillance-driven ML, particularly in settings where genomic sequencing and detailed clinical data are not routinely available.

### Public health implications

The framework developed in this study has several potential applications for AMR surveillance and AMS. First, surveillance-derived prediction models could complement conventional surveillance systems by providing early estimates of antimicrobial susceptibility while laboratory testing is still in progress. Although prospective clinical evaluation is required before routine implementation, such predictions could support AMS and inform empirical treatment decisions pending laboratory confirmation. Secondly, integrating predictive analytics into surveillance programmes could enhance existing descriptive surveillance by identifying geographical variation in susceptibility patterns and generating hypotheses regarding emerging resistance trends. Such information could also strengthen AMS programmes, surveillance prioritisation, and resource allocation. Thirdly, because the framework relies exclusively on routinely collected surveillance metadata, it has the potential to be implemented within existing surveillance infrastructures without requiring widespread genomic sequencing capacity. This characteristic is particularly relevant for LMICs, where surveillance systems frequently collect epidemiological metadata but have limited access to advanced molecular diagnostics. Although the present study focused on methodological development rather than implementation, the framework illustrates one pathway by which surveillance programmes could evolve from retrospective reporting towards data-driven predictive surveillance.

## Limitations

Several limitations should be considered when interpreting the findings of this study.

This was a retrospective secondary analysis of routinely collected surveillance data. Although the Pfizer ATLAS programme provides one of the largest standardized antimicrobial susceptibility datasets currently available, surveillance data are not collected through population-based sampling and may therefore be subject to selection bias. Participating laboratories are predominantly tertiary referral centres and larger healthcare facilities, which may limit the representativeness of resistance patterns observed within the wider population. Also, model development was intentionally restricted to African isolates collected between 2019 and 2023 to produce a proof-of-concept framework relevant to LMICs. While this improves regional applicability, it may limit the generalisability of the models to other geographic regions with different epidemiological characteristics, antimicrobial prescribing practices, and resistance profiles. External validation using independent surveillance datasets from other settings is therefore required before broader implementation can be considered.

Although class imbalance was addressed during model development using RandomOverSampler applied exclusively to the training dataset, substantial imbalance remained within several antibiotic-specific datasets. Consequently, prediction performance for minority susceptibility classes, particularly intermediate and resistant isolates, was generally lower than for susceptible isolates. Overall accuracy should therefore be interpreted alongside class-specific performance metrics. Furthermore, the predictor variables were intentionally limited to routinely collected surveillance metadata. Important patient-level variables, including previous antimicrobial exposure, comorbidities, healthcare-associated risk factors, prior hospitalization, treatment history, and clinical outcomes, were unavailable within the dataset and therefore could not be incorporated into the prediction models. Similarly, genomic determinants of AMR were not included. Although this design improves the practicality of the framework for routine surveillance, incorporating these variables may further improve predictive performance in future studies.

Temporal changes in AMR were also not explicitly modelled. Although recent isolates (2019-2023) were selected to improve epidemiological relevance, resistance patterns continue to evolve. Periodic model retraining and future incorporation of longitudinal or time-series modelling approaches may therefore be necessary to maintain predictive performance. Although susceptibility interpretations used for model development were based on the CLSI-derived categories provided within the downloaded ATLAS dataset, temporal changes in breakpoint definitions and laboratory practices may have introduced residual heterogeneity. Restricting the modelling period reduced, but did not eliminate, this potential source of variation.

Finally, model calibration and prospective clinical performance were not evaluated. Although the models demonstrated encouraging performance on an independent hold-out test dataset, additional evaluation is required to determine the reliability of predicted probabilities and their performance within real-world clinical workflows. The interactive dashboard developed in this study should be regarded as a research prototype. No formal usability assessment, implementation study, or clinical impact evaluation was undertaken, and the dashboard should therefore not be interpreted as a validated clinical decision-support system. Future research should prioritise external validation using independent surveillance datasets, prospective evaluation within routine clinical workflows, assessment of model calibration and clinical utility, incorporation of longitudinal resistance trends and additional clinical or genomic predictors, and formal usability and implementation studies to determine how predictive surveillance tools can best support antimicrobial stewardship and public health decision-making.

## Conclusion

AMR remains a major global public health challenge, underscoring the need for surveillance systems that not only describe existing resistance patterns but also provide earlier insights into emerging threats. This study demonstrates that routinely collected AST surveillance data can be used to develop antibiotic-specific ML models across seven clinically important antibiotics without requiring genomic sequencing or detailed patient-level clinical information.

By combining microbiological, epidemiological, and geographic surveillance variables with optimized XGBoost models and an interactive visualization platform, this study illustrates how routinely collected surveillance data can be transformed into a foundation for surveillance-driven predictive analytics. The findings demonstrate that existing surveillance data contain meaningful predictive information that could support the development of predictive approaches for AMR surveillance and AMS, particularly in resource-limited settings.

Although the proposed framework should currently be regarded as a proof of concept, future work should focus on external validation, model calibration, prospective clinical evaluation, incorporation of additional clinical and genomic predictors, and assessment of implementation within routine surveillance systems. Collectively, this study provides a reproducible foundation for the continued development and evaluation of predictive antimicrobial resistance surveillance tools that complement conventional surveillance approaches and support earlier, evidence-informed responses to emerging resistance threats.

## Mathematical scripts

No explicit mathematical equations were used. All modeling was implemented using standard machine learning algorithms (XGBoost) in Python.

## Ethics and consent

Ethical approval and consent were not required. Ethical approval was not required for this study, as it used secondary, de-identified data from the Pfizer ATLAS database.

## Data Availability

The antimicrobial susceptibility data used in this study were obtained from the Pfizer ATLAS program. Access to this dataset is available through the Vivli data sharing platform, subject to data access agreements, using the following information: Data contributor name: Pfizer (AMR); DOI: https://doi.org/10.25934/PR00007930; AMR ID: VIV00007930; and Dataset ID: ATLAS_Antibiotics. The interpretive model was trained using MIC breakpoints and guidelines in the 35th edition of the CLSI 2025 standards ( Clinical and Laboratory Standards Institute, 2025). No new primary data were generated in this study. No extended data are associated with this article.

## Software availability

Source code available from:

https://github.com/davidgichohi/amr-predictor

https://github.com/davidgichohi/AMR-MIC-1

Archived software available from:

https://doi.org/10.5281/zenodo.19681458

https://doi.org/10.5281/zenodo.19681397

License:

MIT License.

Software environment: All data management, preprocessing, statistical analyses, and machine learning model development were performed in Python 3.10 using reproducible Jupyter Notebook workflows. Data manipulation was conducted using pandas and NumPy, while data visualisation was performed using Matplotlib. Machine learning models were developed using XGBoost and scikit-learn, with class imbalance addressed using imbalanced-learn and hyperparameter optimisation performed using GridSearchCV. The rule-based MIC interpretation tool and interactive dashboard were implemented using the Flask web framework.
