# Supplementary material for: Surveillance-Driven Machine Learning for Prediction of Antimicrobial Susceptibility: An Explainable Modeling Framework using the Pfizer ATLAS Dataset (2004 - 2023)
Source: Wellcome Open Res. 2026 Sep 21;11:393. Originally published 2026 Jun 27. [Version 2] doi: 10.12688/wellcomeopenres.26477.2 (PMC13624498; doi:10.12688/wellcomeopenres.26477.2)
Supplement: Peer review history

# Reviewer report: Reviewer response for version 2

**DOI:** 10.21956/wellcomeopenres.30447.r169686

**Published:** 2026-09-29

**Authors:** Daniel Edbert

**Affiliations:** 1 Atma Jaya Catholic University of Indonesia, Jakarta, Indonesia

This manuscript version already presents a practical approach to addressing a critical healthcare challenge in resource-limited settings: predicting antimicrobial susceptibility before laboratory phenotypic AST results become available. 

 The inclusion of an open-source analytical pipeline, an MIC rule-based interpretation tool, and an interactive prototype dashboard demonstrates a clear vision for translation and reproducibility.

 However, certain minor revisions should be put into concern:

 In the main manuscript title, the authors wrote a date range spanning 2004–2023, whereas in the Abstract and Methods sections, they clarified that predictive modeling was restricted to isolates from 2019–2023. While the problem is that a discrepancy between the title and the methodology creates confusion regarding the exact cohort scope used to train and validate the machine learning algorithms, the authors should consider updating the title to explicitly reflect the 2019–2023 dataset cohort analyzed in the modeling pipeline.

 In the Abstract, Results, Table 1, and Discussion, the authors wrote model evaluations relying primarily on overall test accuracy values (e.g., 57%–76%). While the problem is that overall accuracy can be misleading in imbalanced biological datasets by masking poor performance on critical minority classes, the authors should consider replacing or supplementing accuracy values with a comprehensive table reporting macro/weighted F1-scores, class-specific precision/recall, and specifically Resistant (R) class recall across all seven antibiotics.

 In the Methods under *Training predictive machine learning models*, the authors wrote that RandomOverSampler was applied exclusively to the training dataset. While the problem is that naive oversampling on tabular data creates exact duplicate feature vectors that can cause XGBoost to overfit, the authors should consider providing a clear rationale for selecting RandomOverSampler and discussing how overfitting was mitigated.

 In the Discussion, the authors wrote general interpretations of performance variations without examining class-specific sensitivity. While the problem is that failing to evaluate resistant strain, identification leaves clinical safety unaddressed, the authors should consider expanding the narrative to examine how reliably models identify resistant strains and discussing the clinical risk of misclassifying a resistant pathogen as susceptible.

Is the work clearly and accurately presented and does it cite the current literature?

Yes

If applicable, is the statistical analysis and its interpretation appropriate?

Partly

Are all the source data underlying the results available to ensure full reproducibility?

Partly

Is the study design appropriate and is the work technically sound?

Yes

Are the conclusions drawn adequately supported by the results?

Partly

Are sufficient details of methods and analysis provided to allow replication by others?

Partly

Reviewer Expertise:

Antimicrobial resistance, Microbiology, Infectious Disease

I confirm that I have read this submission and believe that I have an appropriate level of expertise to confirm that it is of an acceptable scientific standard.

# Reviewer report: Reviewer response for version 1

**DOI:** 10.21956/wellcomeopenres.29157.r159850

**Published:** 2026-07-20

**Authors:** Olga Perovic

**Affiliations:** 1 National Institute for Communicable Diseases, Johannesburg, South Africa

Surveillance-Driven Machine Learning for Prediction of Antimicrobial Susceptibility for reasoning and approach presents the main findings, methods, and implications of the research article, focusing on how machine learning models were developed using surveillance data to predict antimicrobial susceptibility. Antimicrobial resistance (AMR) is a critical global health issue, particularly in Sub-Saharan Africa, due to limited diagnostic infrastructure and stewardship programs. Traditional antimicrobial susceptibility testing (AST) is slow (48–72 hours), often leading to empirical use of broad-spectrum antibiotics, which implicates in development of resistance. The study aimed to develop and evaluate explainable machine learning models that predict antibiotic susceptibility using routinely collected surveillance data, specifically from the Pfizer ATLAS dataset (2004–2023). The focus was on recent African isolates (2019–2023) to ensure regional relevance. Pfizer ATLAS global AMR surveillance dataset, filtered for African isolates from 2019–2023. They developed rule-based tools to interpret Minimum Inhibitory Concentration (MIC) values and trained antibiotic-specific models to predict susceptibility with epidemiological details. Assessed models using accuracy, precision, and recall, to create an interactive web-based dashboard for data visualization and decision support. Testing accuracies was in range from mid to high for antibiotics. Bacterial species/family and geographic location were the strongest predictors; demographic variables were less predictive. Interpretability: analysis showed that species, family, and location most influenced predictions. Dashboard enabled users to explore AMR trends, interpret MIC values, and predict antibiotic effectiveness interactively. The models can support earlier, more informed antibiotic selection, especially where rapid diagnostics are unavailable. For public health perspective predictive models can enhance surveillance, inform stewardship programs, and guide resource allocation. The approach is compatible with existing surveillance infrastructure and does not require advanced genomic data, making it suitable for low- and middle-income countries.

 Limitations

 1. Data bias: Surveillance data may overrepresent tertiary/urban centers.

 2.Class imbalance: More susceptible than resistant isolates, affecting model performance. 3. Missing clinical data: Lack of detailed patient-level information limits individualized predictions.

 4.Temporal pattern: Resistance patterns change over time; models need regular updating.

 5. Reproducibility: Peer review noted incomplete computational reproducibility and called for more detailed sharing of scripts, models, and processed data.

 The study demonstrates that routinely collected AMR surveillance data can be transformed into interpretable, scalable predictive tools for antimicrobial susceptibility. These tools can support empirical prescribing and stewardship, particularly in resource-limited settings. However, further validation, improved reproducibility, and integration of additional clinical variables are needed before routine clinical adoption.

 Overall, the manuscript is well-organized and relevant but should provide more methodological detail and a more cautious interpretation of clinical applicability. Reproducibility and open-source need stronger evidence. The dashboard's scientific contribution requires formal evaluation.

Is the work clearly and accurately presented and does it cite the current literature?

Yes

If applicable, is the statistical analysis and its interpretation appropriate?

Yes

Are all the source data underlying the results available to ensure full reproducibility?

Partly

Is the study design appropriate and is the work technically sound?

Yes

Are the conclusions drawn adequately supported by the results?

Yes

Are sufficient details of methods and analysis provided to allow replication by others?

Yes

Reviewer Expertise:

AMR program including surveillance and quality methods in laboratory setting.

I confirm that I have read this submission and believe that I have an appropriate level of expertise to confirm that it is of an acceptable scientific standard.

# Response

**Published:** 2026-08-30

**Authors:** Raphael Mutua

**Affiliations:** Center for Microbiology Research, Kenya Medical Research Institute, Nairobi, Nairobi County, Kenya

We sincerely thank the reviewer for the careful assessment of our manuscript and for recognising the relevance of the study to antimicrobial resistance surveillance, particularly in resource-limited settings. We appreciate the positive evaluation of the study design, analytical approach, and overall scientific contribution. We have also carefully considered the limitations and recommendations highlighted in the report and revised the manuscript accordingly. **Comment 1: Data representativeness and potential surveillance bias** *Surveillance data may overrepresent tertiary or urban centres.*

**Response:** We agree that the Pfizer ATLAS dataset may not be fully representative of routine clinical populations in the included countries. Participating centres may disproportionately reflect tertiary, urban, or better-resourced facilities, and country-level contributions were uneven. The Discussion has therefore been expanded to acknowledge potential site-selection, referral, testing, and sampling biases. We also clarify that the findings should not be interpreted as population-representative estimates and that external validation is required before broader generalisation.

**Comment 2: Class imbalance** *More susceptible than resistant isolates may affect model performance.*

**Response:** We thank the reviewer for highlighting this important limitation. The revised Methods now provide a more detailed explanation of the class-imbalance strategy. RandomOverSampler was applied exclusively to the training dataset, while the independent test dataset retained the original susceptibility distribution to prevent data leakage. The Results and Discussion now also emphasise that performance was generally stronger for susceptible isolates and weaker for intermediate and resistant classes. We acknowledge that class imbalance remained an important limitation despite the use of oversampling.

**Comment 3: Limited patient-level clinical data** *The lack of detailed clinical information limits individualised prediction.*

**Response:** We agree. The models were intentionally developed using routinely collected surveillance variables to evaluate feasibility in settings where comprehensive electronic health records and genomic data may be unavailable. However, this design limits patient-specific prediction and does not capture important clinical factors such as prior antimicrobial exposure, comorbidities, recent hospitalisation, treatment history, or previous microbiological results. This limitation is now more explicitly discussed, and the inclusion of richer clinical variables is identified as a priority for future model development.

**Comment 4: Temporal changes in resistance** *Resistance patterns change over time and models require regular updating.*

**Response:** We agree that temporal drift is an important consideration. The analysis was restricted to African isolates collected between 2019 and 2023 to improve contemporary relevance and reduce the influence of older resistance patterns. Nevertheless, resistance epidemiology, antimicrobial use, laboratory practices, and clinical breakpoints may change over time. The revised Discussion now states that the models would require periodic reassessment, recalibration, and retraining before any prospective or routine application. **Comment 5: Computational reproducibility** *More detailed sharing of scripts, models, and processed data is required.*

**Response:** We appreciate this observation. The Methods have been expanded to provide a clearer description of data preprocessing, predictor encoding, class balancing, data partitioning, hyperparameter optimisation, model evaluation, and feature-importance analysis. We have also moderated claims regarding reproducibility and open-source availability where the supporting computational resources are incomplete. We acknowledge that provision of the complete preprocessing pipeline, trained models, breakpoint mappings, feature encodings, and source code would further improve reproducibility.

**Comment 6: Clinical applicability and dashboard evaluation** *Further validation is required before clinical adoption, and the dashboard requires formal evaluation.*

**Response:** We agree. The dashboard is now consistently described as a research prototype intended to illustrate the visualisation and exploration of surveillance-derived outputs. The manuscript explicitly states that no usability testing, implementation study, or clinical validation was conducted and that the dashboard should not be interpreted as a validated clinical decision-support system. Claims regarding empirical prescribing, antimicrobial stewardship, and clinical utility have also been moderated throughout the manuscript. The framework is now presented as a proof of concept requiring external validation, calibration assessment, prospective evaluation, and implementation research before clinical use. We sincerely thank the reviewer for the positive assessment and constructive recommendations. The comments helped us strengthen the manuscript’s treatment of dataset representativeness, class imbalance, temporal changes, reproducibility, and the limits of clinical applicability. We believe the revised manuscript now presents the findings and limitations more clearly and cautiously.

# Reviewer report: Reviewer response for version 1

**DOI:** 10.21956/wellcomeopenres.29157.r159846

**Published:** 2026-07-20

**Authors:** Timothy Miles Rawson

**Affiliations:** 1 Imperial College London, London, UK

**General Comments**

 This manuscript addresses an important and timely topic: whether routinely collected antimicrobial resistance (AMR) surveillance metadata can be leveraged to predict antimicrobial susceptibility using machine learning (ML) approaches. The use of the Pfizer ATLAS dataset, the focus on explainable modelling, and the intention to develop open-source tools are all potentially valuable contributions to the AMR and surveillance communities.

 The study had several objectives, including: (1) development of a rule-based MIC-to-susceptibility interpretation tool, (2) development of an ML model to predict susceptibility from surveillance metadata, (3) evaluation of model performance, (4) identification of key predictors, and (5) provision of an open-source implementation.

 These are worthwhile aims and, if executed rigorously, could provide useful methodological advances.

 However, in its current form, the manuscript lacks sufficient methodological detail and clarity for the work to be adequately assessed.

 Many aspects of the study design, data processing, model development, and evaluation are either absent or insufficiently described. As a result, it is difficult to determine the validity, reproducibility, and generalisability of the findings.

*Substantial revision is therefore required before it is possible to understand the methods used and assess the validity, reproducibility, and generalisability of the findings.*

**Major Comments**

**1. Insufficient methodological reporting**

 The Methods section lacks the detail required to understand, reproduce, or critically assess the study. Given that this is a prediction modelling study, the authors should follow established reporting standards such as the TRIPOD guidelines for predictive modelling studies.

 Specifically, the manuscript should clearly describe:

- Data extraction and inclusion/exclusion criteria.
- Organisms included in the analysis.
- Antimicrobials included and rationale for their selection.
- Data cleaning and pre-processing procedures.
- Handling of missing data.
- Feature engineering procedures.
- Model development workflow.
- Training, validation, and testing procedures.
- Performance metrics and statistical analyses.

Currently, several of these elements are either missing entirely or appear only briefly in the Results section.

**2. Lack of clarity regarding susceptibility testing methods**

 The ATLAS dataset contains susceptibility data generated using different interpretive frameworks and laboratory methodologies over time. The manuscript notes the use of MIC data but does not adequately explain how differences in susceptibility testing standards were handled.

 In particular:

- Were CLSI and EUCAST breakpoints both present within the dataset?
- How were differences between laboratory methods and breakpoint systems managed?
- Was the interpretive standard included as a model feature?
- Were analyses stratified by breakpoint methodology?
- Could changes in breakpoint definitions over time have influenced model performance?

These issues are potentially important sources of bias and must be addressed explicitly.

**3. Rule-based MIC interpretation tool is insufficiently described**

 One of the stated objectives is development of a rule-based tool to convert MIC values into susceptibility categories. However, there is very little detail describing:

- The underlying decision rules.
- Which breakpoint standards were used (CLSI, EUCAST, or both).
- How discrepancies between breakpoint systems were handled.
- Whether breakpoint updates over time were considered.
- How the tool was validated.

The manuscript also does not clearly explain how this interpretive tool was applied within the modelling workflow. A more detailed description is required.

**4. Machine learning model development lacks transparency**

 The ML methodology is not described in sufficient detail.

 The manuscript should clearly specify:

- Which algorithms were developed and evaluated.
- Hyperparameter optimisation procedures.
- Feature selection methods.
- Data partitioning strategies.
- Internal validation approaches.
- Measures taken to prevent data leakage.

Training and testing datasets are referenced in the Results section but are not adequately described in the Methods section. This information is fundamental to evaluating model performance.

**5. Unclear specification of predictor variables**

 The manuscript refers to predictor variables and key features but provides limited information regarding the actual metadata used.

 For example, phrases such as "among others" are not sufficiently informative in a scientific manuscript.

 The authors should provide a complete description of:

- All candidate predictor variables.
- Variables ultimately included in the final model.
- Their definitions and sources.
- The rationale for their inclusion.

It would also be helpful to know whether laboratory testing methodology was included as a predictor variable.

**6. Representativeness and generalisability of the ATLAS dataset**

 The manuscript should provide a more critical discussion of the representativeness of ATLAS data and the extent to which findings are generalisable.

 For example:

- How representative are the included isolates of routine clinical populations?
- Are participating centres evenly distributed geographically?
- If analyses focus primarily on African datasets, to what extent can models be generalised beyond those settings?
- Could surveillance or sampling biases influence model performance?

These considerations are important when interpreting claims regarding broader applicability.

**7. Justification of organism and susceptibility test selection**

 The manuscript does not sufficiently describe:

- Which organisms were included.
- Why those organisms were selected.
- Why only a relatively small number of susceptibility tests appear to have been used.

A clearer justification is needed, particularly given the stated aim of developing broadly applicable predictive models.

**Comments on Results**

 The Results section currently lacks sufficient detail to permit meaningful evaluation.

 Specifically:

- Model performance metrics are not described in enough detail.
- Confidence intervals and uncertainty estimates should be provided.
- Comparative performance across organisms, antimicrobials, and geographic regions would strengthen interpretation.
- It remains unclear whether susceptibility testing methodology was included as a predictor variable and whether its inclusion affected performance.
- Additional information on feature importance and explainability outputs is required.

**Conclusion**

 This study addresses an important problem with potentially substantial relevance to antimicrobial resistance surveillance and clinical microbiology. The use of large-scale surveillance datasets and explainable machine learning approaches is promising. However, the manuscript in its current form lacks the methodological transparency and reporting detail required for proper scientific evaluation & determination of whether the authors primary aims were met.

 I encourage the authors to substantially revise the manuscript, particularly the Methods section, following established prediction-model reporting standards such as TRIPOD. Greater clarity regarding data handling, breakpoint interpretation, predictor variables, model development, validation procedures, and dataset representativeness will be essential before the manuscript can be adequately assessed. At present, the methodological omissions are sufficiently substantial that the study is not yet suitable for detailed review.

 With substantial revision and improved reporting, the work has the potential to make a useful contribution to the field.

Is the work clearly and accurately presented and does it cite the current literature?

No

If applicable, is the statistical analysis and its interpretation appropriate?

No

Are all the source data underlying the results available to ensure full reproducibility?

Yes

Is the study design appropriate and is the work technically sound?

No

Are the conclusions drawn adequately supported by the results?

Partly

Are sufficient details of methods and analysis provided to allow replication by others?

No

Reviewer Expertise:

Infectious Disease, Medical Microbiology, Machine Learning, Antimicrobial Susceptibility Testing

I confirm that I have read this submission and believe that I have an appropriate level of expertise to state that I do not consider it to be of an acceptable scientific standard, for reasons outlined above.

# Response

**Published:** 2026-08-30

**Authors:** Raphael Mutua

**Affiliations:** Center for Microbiology Research, Kenya Medical Research Institute, Nairobi, Nairobi County, Kenya

We sincerely thank the reviewer for the careful and detailed assessment of our manuscript. We appreciate the reviewer’s recognition of the importance of the research question and the potential value of using routinely collected antimicrobial resistance surveillance data for predictive modelling. We also acknowledge that the original manuscript did not provide sufficient methodological detail to permit a full assessment of the study’s validity, reproducibility, and generalisability. In response, we have extensively revised the manuscript, with particular emphasis on the Methods, Results, Discussion, and Conclusion. The revised version now provides a clearer account of data selection, preprocessing, breakpoint interpretation, predictor variables, model development, internal validation, class-imbalance management, hyperparameter optimisation, performance evaluation, model interpretability, and study limitations. We have also moderated claims regarding clinical application and reframed the dashboard as a research prototype rather than a validated decision-support system. Our point-by-point responses are provided below.

**Comment 1: Insufficient methodological reporting** *The Methods section lacks the detail required to understand, reproduce, or critically assess the study. Given that this is a prediction modelling study, the authors should follow established reporting standards such as the TRIPOD guidelines for predictive modelling studies.* *Specifically, the manuscript should clearly describe data extraction and inclusion/exclusion criteria, organisms and antimicrobials included, data cleaning and preprocessing, missing data, feature engineering, model development, data partitioning, validation, performance metrics, and statistical analyses.* **Response** We agree that the original Methods section did not provide sufficient detail regarding the prediction-model development workflow. The Methods have now been comprehensively expanded and reorganised. The revised manuscript describes:

- the retrospective study design and Pfizer ATLAS data source;
- restriction of the analysis to African isolates collected between 2019 and 2023;
- inclusion of organisms listed in the WHO Bacterial Priority Pathogens List;
- criteria used to select antibiotics for model development;
- exclusion of records with missing predictor or outcome information;
- exclusion of variables with extensive missingness or no informative observations;
- the complete set of candidate predictor variables;
- one-hot encoding of categorical predictors;
- label encoding of susceptibility outcomes;
- stratified 80:20 training and hold-out test partitioning;
- restriction of RandomOverSampler to the training dataset to prevent data leakage;
- hyperparameter optimisation using GridSearchCV and three-fold stratified cross-validation;
- the complete hyperparameter search grid and model-selection criterion;
- retraining of the selected model on the balanced training data;
- evaluation on an independent, unaltered test dataset; and
- the performance metrics and feature-importance approach used.

We also revised the organisation of the manuscript so that methodological information previously appearing only in the Results is now presented in the Methods. These changes were informed by established prediction-model reporting principles, including the need to distinguish clearly between model development, internal validation, and independent test evaluation.

**Comment 2: Lack of clarity regarding susceptibility testing methods** *The ATLAS dataset contains susceptibility data generated using different interpretive frameworks and laboratory methodologies over time. The manuscript does not adequately explain how differences in susceptibility testing standards were handled.* *Were CLSI and EUCAST breakpoints both present? How were differences between laboratory methods and breakpoint systems managed? Was the interpretive standard included as a model feature? Were analyses stratified by breakpoint methodology? Could changes in breakpoint definitions over time have influenced model performance?*

**Response** We have revised the Methods to clarify the susceptibility interpretations used in the study. The Pfizer ATLAS programme reports MIC measurements together with standardised susceptibility interpretations derived using Clinical and Laboratory Standards Institute breakpoints. Although the ATLAS platform also permits users to explore susceptibility using European Committee on Antimicrobial Susceptibility Testing breakpoints, the downloaded dataset used for the present analysis contained the standardised CLSI-derived susceptibility categories. All model outcomes in this study were therefore based on those CLSI-derived categories. Because a single interpretive framework was used for the analysed outcomes, CLSI and EUCAST categories were not combined or harmonised, breakpoint methodology was not included as a predictor, and analyses were not stratified by interpretive system. We have also clarified that the study was restricted to isolates collected between 2019 and 2023 partly to improve contemporary epidemiological relevance and reduce the influence of older resistance patterns. We nevertheless acknowledge that breakpoint definitions and laboratory practices may change over time and that residual heterogeneity in multicentre surveillance data remains a possible source of bias. This issue is now recognised in the discussion of dataset limitations and generalisability.

**Comment 3: Rule-based MIC interpretation tool is insufficiently described** *One of the stated objectives is development of a rule-based tool to convert MIC values into susceptibility categories. However, there is very little detail describing the underlying decision rules, breakpoint standards, handling of discrepancies, breakpoint updates, validation, and how the tool was used within the modelling workflow.*

**Response** The description of the rule-based MIC interpretation tool has been expanded substantially. The revised Methods now clarify that the tool did not derive new clinical breakpoints. Instead, it operationalised the CLSI-derived susceptibility interpretations contained within the Pfizer ATLAS dataset. For each organism-antibiotic combination, MIC values and their corresponding susceptibility categories were extracted and represented as structured JavaScript Object Notation lookup rules. Within the dashboard, the user selects the organism and antibiotic and enters an MIC value, after which the application returns the corresponding susceptible, intermediate, or resistant category. We have also clarified that the rule-based component and the machine-learning component perform different functions. The rule-based tool translates an observed MIC into a susceptibility category, whereas the machine-learning models predict susceptibility from routinely collected epidemiological and isolate-level metadata when MIC information is not used as a predictor. No formal independent validation of the dashboard-based interpretive tool was conducted. We therefore revised the manuscript to describe it as a research prototype that demonstrates the operationalisation of existing interpretations rather than as a newly validated diagnostic or clinical tool. The manuscript now explicitly states that it should not replace established laboratory AST interpretation procedures.

**Comment 4: Machine-learning model development lacks transparency** *The manuscript should clearly specify which algorithms were developed and evaluated, hyperparameter optimisation procedures, feature selection methods, data partitioning strategies, internal validation approaches, and measures taken to prevent data leakage.*

**Response** We agree that the original description of the model-development process was insufficient. The revised Methods now provide a detailed account of the modelling workflow. We clarify that Random Forest and baseline XGBoost approaches were explored during model development. The final models were antibiotic-specific XGBoost classifiers. XGBoost was selected because of its suitability for structured tabular data and its capacity to represent nonlinear relationships and interactions among categorical surveillance variables. For each antibiotic, observations were divided into stratified training and testing sets using an 80:20 split. RandomOverSampler was applied only to the training set to address class imbalance, while the test set retained the original class distribution. This separation was used to prevent synthetic or duplicated training observations from entering the test dataset. Hyperparameter optimisation was performed using GridSearchCV with three-fold stratified cross-validation. The search grid included maximum tree depths of 3, 5, and 7; 50 and 100 boosting trees; learning rates of 0.05 and 0.10; and subsampling fractions of 0.8 and 1.0. The model achieving the highest weighted F1-score across the cross-validation folds was selected and retrained on the balanced training set before evaluation on the independent test set. No separate feature-selection procedure was applied. All prespecified routinely collected surveillance predictors that met data-quality requirements were retained. This has now been stated explicitly.

**Comment 5: Unclear specification of predictor variables** *The manuscript refers to predictor variables and key features but provides limited information regarding the actual metadata used. The authors should provide a complete description of all candidate predictors, variables included in the final model, their definitions and sources, and the rationale for their inclusion.*

**Response** The revised Methods now provide a complete list of the candidate and final predictor variables used in the antibiotic-specific models:

- bacterial species;
- bacterial family;
- country of isolation;
- patient sex;
- age group;
- clinical specialty; and
- specimen source.

These variables were obtained directly from the Pfizer ATLAS surveillance dataset. They were selected because they are routinely recorded in microbiological surveillance systems and therefore support the study’s aim of evaluating a modelling framework that does not depend on genomic sequencing or detailed electronic health-record data. All predictors were categorical and were converted into numerical representations using one-hot encoding. No numerical scaling or normalisation was required. Laboratory testing methodology and interpretive standard were not included as predictor variables because the model outcomes used the standardised CLSI-derived susceptibility classifications supplied in the analysed dataset. We have removed nonspecific wording such as “among others” and now state the full predictor set consistently throughout the manuscript.

**Comment 6: Representativeness and generalisability of the ATLAS dataset** *The manuscript should provide a more critical discussion of the representativeness of ATLAS data and the extent to which findings are generalisable.*

**Response** We agree that the representativeness of the dataset and the limits of generalisability required greater attention. The revised Discussion now explains that ATLAS is a multicountry surveillance programme based on participating centres and should not be interpreted as a population-representative sample of all infections occurring within the included countries. We acknowledge that participating laboratories may disproportionately represent tertiary, urban, or better-resourced facilities and that countries and centres were not evenly represented. South Africa, Nigeria, and Morocco contributed the largest numbers of observations in the analytical dataset. Referral patterns, local testing practices, isolate selection, healthcare access, and site participation may therefore influence the observed susceptibility distributions and model performance. We also clarify that the models were intentionally developed using recent African isolates as a regional proof of concept. Their performance should not be assumed to generalise automatically to other geographic regions, healthcare settings, time periods, or laboratory networks. External validation in independently collected and appropriately harmonised datasets is required before broader application. Claims regarding broad applicability have consequently been moderated throughout the Introduction, Discussion, and Conclusion.

**Comment 7: Justification of organism and susceptibility-test selection** *The manuscript does not sufficiently describe which organisms were included, why those organisms were selected, and why only a relatively small number of susceptibility tests appear to have been used.*

**Response** The revised Methods now state that the analysis was restricted to bacterial species included in the WHO Bacterial Priority Pathogens List because these organisms represent major priorities for global AMR surveillance and public-health action. The revised Results describe the principal organisms represented in the analytical dataset, including *Escherichia coli*, *Klebsiella pneumoniae*, *Pseudomonas aeruginosa*, *Staphylococcus aureus*, and *Acinetobacter baumannii*. Antibiotic-specific models were developed for seven antibiotics with sufficient susceptibility observations to support model training and hold-out evaluation: gentamicin, ceftazidime-avibactam, amikacin, imipenem, piperacillin-tazobactam, ceftolozane-tazobactam, and ceftazidime. Antibiotic selection was therefore driven by data availability and suitability for model development rather than an intention to provide comprehensive coverage of all clinically relevant antimicrobial agents.  

**Comments on the**

**Results** **Result Comment 1: Model performance is insufficiently described** *Model performance metrics are not described in enough detail.*

**Response** The Results have been expanded to distinguish training from hold-out test performance across the seven antibiotic-specific models and to describe variation in predictive performance between antibiotics. Training accuracy ranged from 73% to 86%, while test accuracy ranged from 57% to 76%. The Methods now specify accuracy, precision, recall, F1-score, and confusion matrices as the evaluation measures used in the modelling workflow. The revised Results also interpret model performance in the context of the substantial class imbalance observed across the antibiotic-specific datasets. Because class-specific precision, recall, and F1-score values were not retained as complete numerical outputs for all seven definitive models, these metrics are not tabulated in the main manuscript.  

**Result Comment 2: Confidence intervals and uncertainty estimates** *Confidence intervals and uncertainty estimates should be provided.*

**Response** We agree that uncertainty estimates would strengthen the interpretation of model performance. Confidence intervals were not generated during the original model-development workflow, and we have not retrospectively introduced estimates that were not produced by the analysis. We have therefore avoided overstating differences between models and now present the reported performance measures descriptively. The absence of confidence intervals and resampling-based uncertainty estimates is acknowledged as a limitation. Future evaluations should use approaches such as bootstrap resampling or repeated cross-validation to quantify uncertainty around performance metrics. Because this request was not fully addressed analytically, we have stated this transparently rather than implying that confidence intervals were calculated.  

**Result Comment 3: Comparative performance across organisms, antimicrobials, and regions** *Comparative performance across organisms, antimicrobials, and geographic regions would strengthen interpretation.*

**Response** We thank the reviewer for this suggestion. The revised Results provide clearer comparisons across antibiotic-specific models, including variation in training and testing accuracy and the influence of class imbalance and sample size. However, separate models stratified by organism and geographic region were not developed as part of the original analytical plan. Conducting such subgroup analyses with the present data could also produce unstable estimates where organism-country-antibiotic combinations contain limited observations. We have therefore not added post hoc subgroup performance claims that were not supported by the completed analysis. Instead, the revised Discussion identifies organism-specific and geographic validation as priorities for future work and cautions against assuming uniform performance across countries, organisms, or clinical settings.

**Result Comment 4: Susceptibility methodology as a predictor** *It remains unclear whether susceptibility testing methodology was included as a predictor variable and whether its inclusion affected performance.*

**Response** We have clarified that susceptibility-testing methodology and breakpoint standard were not included as model predictors. The analysed outcomes were the standardised CLSI-derived susceptibility categories provided in the downloaded ATLAS dataset. Consequently, no comparison of model performance with and without breakpoint methodology was conducted. This decision and its rationale are now stated explicitly in the Methods.

**Result Comment 5: Feature importance and explainability** *Additional information on feature importance and explainability outputs is required.*

**Response** We thank the reviewer for this valuable suggestion. The revised manuscript now provides additional methodological and interpretive information on model explainability. The Methods specify that predictor importance was quantified using the gain-based feature-importance metric implemented in XGBoost. Gain reflects the improvement in the model objective associated with splits involving a predictor and was used to identify variables contributing most strongly to susceptibility classification. A dedicated Results subsection now reports that bacterial species, bacterial family, and country of isolation were among the most influential predictors, while specimen source and clinical specialty also contributed to prediction. Patient age group and sex generally showed lower relative importance. The Discussion has been expanded to interpret these findings biologically and epidemiologically. Differences between bacterial taxa are plausibly related to intrinsic susceptibility and organism-specific resistance mechanisms, while geographic importance may reflect variation in antimicrobial consumption, healthcare systems, infection-prevention practices, diagnostic capacity, and circulating resistance determinants. We have also clarified the limitations of this approach. Gain-based importance provides a global assessment of predictor contribution but does not explain individual predictions and may be affected by correlations among encoded variables. Future studies should incorporate approaches such as SHAP analysis to provide both global and local explanations of model behaviour.

**Conclusion** *The manuscript lacks the methodological transparency and reporting detail required for proper scientific evaluation. Greater clarity regarding data handling, breakpoint interpretation, predictor variables, model development, validation procedures, and dataset representativeness is essential.*

**Response** We thank the reviewer for this candid and constructive assessment. The manuscript has undergone extensive revision in direct response to these concerns. The Methods now provide a substantially more detailed account of data selection, preprocessing, missing-data handling, predictor encoding, model development, class balancing, hyperparameter optimisation, internal validation, hold-out testing, evaluation metrics, and feature importance. We have clarified the use of CLSI-derived susceptibility categories, expanded the description of the rule-based MIC interpretation component, provided a complete list of predictor variables, and strengthened discussion of ATLAS representativeness and geographic generalisability. The dashboard is now framed solely as a research prototype, and statements regarding clinical utility have been moderated. The revised manuscript consistently presents the framework as a proof of concept requiring external validation, calibration assessment, uncertainty estimation, prospective evaluation, and implementation research before clinical use. We believe these revisions materially improve the transparency and scientific interpretability of the study. We also acknowledge that some limitations remain, particularly the absence of external validation, calibration analysis, confidence intervals, and complete local explainability outputs. These limitations are now stated explicitly rather than being obscured by claims of clinical readiness or broad generalisability. We sincerely thank the reviewer again for the detailed critique. The comments prompted major improvements in the reporting, positioning, and interpretation of the study. We hope that the revised manuscript now provides sufficient information to permit a more complete assessment of its methods, findings, and limitations.

# Reviewer report: Reviewer response for version 1

**DOI:** 10.21956/wellcomeopenres.29157.r159843

**Published:** 2026-07-15

**Authors:** Daniel Edbert

**Affiliations:** 1 Atma Jaya Catholic University of Indonesia, Jakarta, Indonesia

Introduction

 The manuscript is generally well organized and cites recent literature (2024–2026). It effectively places the study within the broader context of surveillance-based AMR prediction and highlights the potential relevance of the proposed approach, particularly for resource-limited settings. The section would be further strengthened by a more critical discussion of previous surveillance-based prediction studies, a clearer articulation of the manuscript's specific novelty, and slightly more cautious framing of the anticipated clinical applications. These revisions would improve the scientific positioning of the study while maintaining the strengths of an already well-written introduction.

 Methods

 The overall concept is appropriate, but important technical weaknesses remain. Model development should add more sufficient methodological rigor (validation strategy, handling of imbalance, hyperparameter optimization details, feature engineering, calibration, external validation). The dashboard itself should not be considered a scientific contribution unless its evaluation is presented. The manuscript states that analyses were version-controlled and containerized, manuscript should insert preprocessing scripts, trained models, JSON breakpoint rules, or complete hyperparameter settings. Replication is currently impossible. the processed datasets, preprocessing pipeline, trained models, feature encodings, breakpoint mappings, and source code should be provided. Therefore, computational reproducibility is incomplete. Several claims (e.g., reproducibility, open-source availability, robustness) should be more demonstrated. Some descriptions are overly promotional rather than evidence-based.

 Results

 Appropriate metrics (accuracy, precision, recall, AUROC) are mentioned in the objectives, but results should mention AUROC values, confidence intervals, confusion matrices, calibration, cross-validation, class-wise performance, and statistical comparisons.

 Discussion

 The Discussion is well organized, clearly written, and demonstrates a good understanding of the current literature on antimicrobial resistance and machine learning. However, the section would benefit from a stronger emphasis on interpreting the study's own findings rather than reiterating general background knowledge. In addition, several statements regarding clinical utility, antimicrobial stewardship, and public health impact should be presented more cautiously, as these applications have not yet been directly evaluated in the current study. Expanding the discussion of the observed model performance, providing a more critical appraisal of methodological limitations (such as external validation, calibration, class imbalance, and generalizability), and aligning the conclusions more closely with the presented evidence would further strengthen the manuscript.

 Conclusion

 The conclusions are directionally supported but occasionally overstate clinical applicability. The conclusion should be put more concise.

Is the work clearly and accurately presented and does it cite the current literature?

Yes

If applicable, is the statistical analysis and its interpretation appropriate?

Partly

Are all the source data underlying the results available to ensure full reproducibility?

Partly

Is the study design appropriate and is the work technically sound?

Yes

Are the conclusions drawn adequately supported by the results?

Partly

Are sufficient details of methods and analysis provided to allow replication by others?

Partly

Reviewer Expertise:

Antimicrobial resistance, Microbiology, Infectious Disease

I confirm that I have read this submission and believe that I have an appropriate level of expertise to confirm that it is of an acceptable scientific standard, however I have significant reservations, as outlined above.

# Response

**Published:** 2026-08-30

**Authors:** Raphael Mutua

**Affiliations:** Center for Microbiology Research, Kenya Medical Research Institute, Nairobi, Nairobi County, Kenya

We sincerely thank the reviewer for the careful evaluation of our manuscript and for the thoughtful and constructive comments. We greatly appreciate the time and expertise devoted to reviewing our work. Your suggestions have substantially improved the clarity, methodological transparency, scientific positioning, and overall balance of the manuscript. We have carefully considered each comment and revised the manuscript accordingly. Our point-by-point responses are provided below.

**Comment 1 - Introduction** *The manuscript is generally well organized and cites recent literature (2024-2026). It effectively places the study within the broader context of surveillance-based AMR prediction and highlights the potential relevance of the proposed approach, particularly for resource-limited settings. The section would be further strengthened by a more critical discussion of previous surveillance-based prediction studies, a clearer articulation of the manuscript's specific novelty, and slightly more cautious framing of the anticipated clinical applications.*

**Response** We thank the reviewer for this positive assessment and helpful suggestions. In response, we substantially revised the Introduction to better position the study within the existing literature. We expanded the discussion of recent surveillance-based antimicrobial susceptibility prediction studies and more clearly distinguished the present work from previous approaches. Specifically, we now emphasise that our study: (i) focuses on African surveillance data as a proof-of-concept for low- and middle-income settings; (ii) develops antibiotic-specific prediction models using routinely collected surveillance metadata without relying on genomic sequencing or detailed patient-level clinical information; (iii) incorporates standardized MIC interpretation within the analytical workflow; and (iv) presents an integrated surveillance-oriented framework combining interpretive and predictive modelling with interactive visualisation. We also moderated statements regarding potential clinical application and consistently describe the framework as a proof of concept requiring prospective validation before routine implementation.

**Comment 2 - Methods** *The overall concept is appropriate, but important technical weaknesses remain. Model development should add more methodological rigor (validation strategy, handling of imbalance, hyperparameter optimization details, feature engineering, calibration, and external validation). The dashboard itself should not be considered a scientific contribution unless its evaluation is presented. The manuscript states that analyses were version-controlled and containerized. Replication is currently impossible. Computational reproducibility is incomplete. Several claims (e.g., reproducibility, open-source availability, robustness) should be more demonstrated. Some descriptions are overly promotional rather than evidence-based.*

**Response** We appreciate the reviewer's detailed and constructive comments regarding methodological transparency. In response, we substantially expanded the Methods section to provide a more comprehensive description of the analytical workflow. The revised manuscript now explicitly describes the study design, inclusion criteria, data preprocessing procedures, handling of missing data, categorical feature encoding, antibiotic selection criteria, train-test data partitioning, management of class imbalance using RandomOverSampler, hyperparameter optimisation using GridSearchCV with three-fold stratified cross-validation, measures implemented to prevent information leakage, model evaluation metrics, and feature importance analysis. We also added explicit discussion of methodological limitations by acknowledging that external validation and formal calibration analyses were not performed because an appropriately harmonised external dataset was unavailable within the timeframe of this study. These limitations are now clearly identified as priorities for future work. Regarding the dashboard, we agree that it should not be presented as evidence of clinical utility without formal evaluation. We have therefore revised the manuscript throughout to describe the dashboard as a **research prototype** developed to demonstrate visualisation and exploration of surveillance-derived predictions. We explicitly state that no usability testing, implementation study, or clinical validation was undertaken and that the dashboard should not be interpreted as a validated clinical decision-support system. With respect to computational reproducibility, we agree that additional resources such as preprocessing scripts, trained models, and complete analytical pipelines would further strengthen reproducibility. Within the scope of this revision, we substantially expanded the methodological descriptions to improve transparency while avoiding claims that exceed the evidence presented. Statements regarding robustness and reproducibility have also been moderated where appropriate.

**Comment 3 - Results** *Appropriate metrics (accuracy, precision, recall, AUROC) are mentioned in the objectives, but results should mention AUROC values, confidence intervals, confusion matrices, calibration, cross-validation, class-wise performance, and statistical comparisons.*

**Response** Following further consideration, we concluded that AUROC was not the most appropriate primary evaluation metric because of the multiclass nature of the prediction task and the marked imbalance between susceptibility categories. Accordingly, we revised both the Methods and Results to explain this decision and now place greater emphasis on class-specific precision, recall, F1-score, and confusion matrices as more informative measures of model performance. The Results section has also been expanded to provide a more detailed description of model development, optimisation, overall predictive performance, and class-wise prediction behaviour. We additionally acknowledge in the Limitations that calibration analyses and external validation were not performed and should be incorporated in future studies. We recognise that confidence intervals and formal statistical comparisons would further strengthen the evaluation and identify these as important areas for future work. **Comment 4 - Discussion** *The Discussion is well organized, clearly written, and demonstrates a good understanding of the current literature on antimicrobial resistance and machine learning. However, the section would benefit from a stronger emphasis on interpreting the study's own findings rather than reiterating general background knowledge. In addition, several statements regarding clinical utility, antimicrobial stewardship, and public health impact should be presented more cautiously.*

**Response** We appreciate the reviewer's thoughtful suggestions. The Discussion has been substantially revised to focus more directly on interpretation of our findings rather than general background information. We expanded the interpretation of observed model performance, discussed the biological plausibility of the identified predictor variables, and considered the influence of factors such as class imbalance, surveillance metadata, and organism-specific resistance patterns on predictive performance. We also moderated statements relating to clinical utility, antimicrobial stewardship, and public health impact. Throughout the revised manuscript, the framework is consistently presented as a proof of concept that demonstrates the feasibility of surveillance-driven prediction rather than a clinically validated decision-support tool. In addition, the limitations section has been expanded to discuss external validation, calibration, class imbalance, generalisability, and the need for prospective implementation studies before routine adoption.

**Comment 5 - Conclusion** *The conclusions are directionally supported but occasionally overstate clinical applicability. The conclusion should be put more concise.*

**Response** The Conclusion has been substantially revised to improve conciseness and to better align with the evidence presented. Statements regarding clinical implementation have been moderated, and the revised conclusion now emphasises that the proposed framework represents a proof of concept demonstrating the potential of routinely collected surveillance data for predictive antimicrobial susceptibility modelling. We also highlight the need for external validation, calibration, and prospective implementation studies before routine clinical application. We sincerely thank the reviewer once again for the constructive feedback and positive assessment of our work. The comments have significantly improved the scientific rigour, transparency, and balance of the manuscript, and we believe the revised version addresses the concerns raised while providing a clearer presentation of the study and its contributions.
